# Scaffold Hopping in Tuberculosis Drug Discovery: Principles, Applications, and Case Studies

**DOI:** 10.1021/acs.jmedchem.5c01100

**Published:** 2025-10-07

**Authors:** Ondrej Kovar, Martin Kufa, Vladimir Finger, Ondrej Soukup, Martin Kratky, Carilyn Torruellas, Jaroslav Roh, Jan Korabecny

**Affiliations:** † Faculty of Pharmacy in Hradec Kralove, 37740 Charles University, Akademika Heyrovskeho 1203, Hradec Kralove 500 03, Czech Republic; ‡ Biomedical Research Center, University Hospital Hradec Kralove, Sokolska 581, Hradec Kralove 500 03, Czech Republic; § Department of Toxicology and Military Pharmacy, Military Faculty of Medicine, University of Defence, Trebesska 1575, Hradec Kralove 500 01, Czech Republic; ∥ U.S. Army CCDC Chemical Biological Center, Aberdeen Proving Ground, Maryland, Maryland 21010-5424, United States

## Abstract

Tuberculosis (TB) imposes a major global health challenge, aggravated by the emergence of drug-resistant *Mycobacterium tuberculosis* (*Mtb*) strains. Scaffold hopping, a medicinal chemistry approach that modifies the molecular backbone of known bioactive compounds, has emerged as a promising tool in the development of novel drugs, including TB therapeutics. This perspective provides an insight into the application of scaffold hopping across varying degrees of structural modifications, highlighting successful case studies targeting key *Mtb* pathways, including energy metabolism, cell wall synthesis, proteasome function, and respiratory processes. Beyond traditional and *in silico* methods, scaffold hopping has spurred the discovery of compounds with improved pharmacological profiles, such as improved pharmacokinetics, enhanced efficacy, reduced toxicity, and resistance circumvention. The findings support scaffold hopping’s potential to address the limitations of current anti-TB drugs as a versatile and innovative approach to accelerate TB drug discovery.

## Graphical abstract

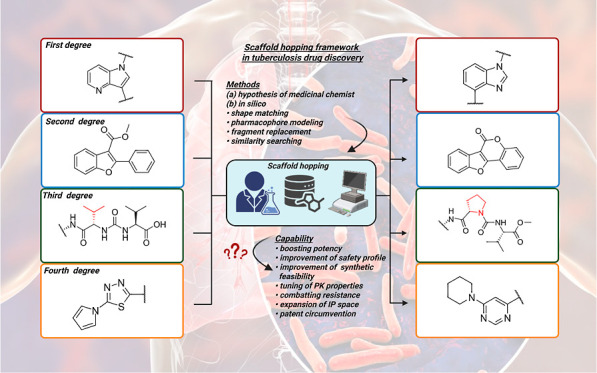

## Introduction

Tuberculosis (TB) is a worldwide infectious bacterial disease caused by strains of *Mycobacterium tuberculosis* (*Mtb*). As one of the most common causes of death from a single infectious agent, the disease represents a major health and economic burden, particularly in sub-Saharan Africa and South-East Asia. According to the 2024 report from the World Health Organization (WHO), there were 10.8 million new cases of *Mtb* infection and approximately 1.25 million TB-related deaths in 2023.^1^ Alarming data from 2023 reveals that approximately 400,000 patients were affected by drug-resistant forms of TB.^1^ In 2015, the WHO set an epidemiological plan, called the “End TB Strategy,” to reduce the number of new TB cases by 80% and deaths by 90% and to mitigate the catastrophic scenario for TB-affected households by 2030.^2^ However, due to unforeseen challenges brought by the COVID-19 pandemic, achieving this goal has been deferred. Although the disease primarily targets the lungs, extrapulmonary forms of TB affecting other tissues also exist (e.g., pleural, CNS, skeletal or miliary TB).^3^ The groups most vulnerable to TB infection and a severe disease course include individuals with compromised immune systems, such as those with HIV/AIDS or more common conditions such as diabetes. It is estimated that up to a quarter of the world́s population is infected with an asymptomatic latent form of TB, which can develop into an acute form throughout a lifetime.^4^

The management strategy for drug-susceptible pulmonary tuberculosis (DS-TB), as recommended in the WHO Module 4 guidelines (2022), involves a 6 month regimen. The therapy begins with the daily administration of isoniazid (INH), rifampicin (RIF), ethambutol (EMB), and pyrazinamide (PZA) for the first two months, followed by four months of INH and RIF therapy. This approach has a success rate of 85%.^5^ Unfortunately, factors such as limited availability of drugs, insufficient level of health care, poor patient compliance, coinfections, or an unhealthy lifestyle can lead to treatment failure. This can result in the emergence of drug-resistant TB (DR-TB), particularly if the infection is not effectively cured. The DR-TB types are classified based on the resistance of *Mtb* strains to individual or combination of drugs used in TB treatment. These include INH-resistant TB (IR-TB), RIF-resistant TB (RR-TB), and multidrug-resistant TB (MDR-TB). MDR-TB is characterized by resistance to both INH and RIF. Pre-extensively drug-resistant TB (pre-XDR-TB) is defined as resistance to the entire class of fluoroquinolones (FQs) or injectable second-line TB therapeutics in addition to RIF and INH, while extensively drug-resistant TB (XDR-TB) is characterized by resistance to the combination of INH, RIF and FQs, along with concurrent resistance to second-line injectable TB therapeutics or newer approved drugs by U.S. Food and Drug Administration (FDA), such as bedaquiline (BDQ), and oxazolidinone-based antibiotics like linezolid (LNZ).^6^ The rise of drug-resistant *Mtb* strains, even against agents not yet widely used,^7–12^ highlights the urgency for innovative drug discovery. Resistance can emerge through mechanisms unrelated to direct drug exposure, such as cross-resistance or intrinsic bacterial adaptability, compromising the effectiveness of future therapies. To address this challenge, the exploration of new chemical entities is crucial, encompassing both the discovery of entirely novel scaffolds and developing more advantageous derivatives from existing anti-TB agents. One such powerful medicinal chemistry tool to achieve this goal is represented by scaffold hopping. By modifying the core structure of known compounds, scaffold hopping enables the design of new candidates with retained or improved activity and optimized properties like solubility, toxicity, and target selectivity.^13–15^ Combining medicinal chemists’ experience and intuition with *in silico* tools,^15^ scaffold hopping has become a versatile and increasingly vital approach for overcoming current drug limitations and expanding therapeutic options against TB.

### Scaffold Hopping

Scaffold hopping is a valuable medicinal chemistry tool frequently involved in the optimization of lead candidates during drug discovery. It refers to the structural modification of the molecular backbone of existing active compounds, leading to the formation of an entirely novel chemotypes. The fundamentals of the scaffold hopping strategy are that structurally distinct compounds can maintain biological activity and affinity for the same biological target if they share key ligand-target interactions as the original molecule.^14,15^ From the medicinal chemist perspective, this approach offers the unique possibility to address various shortcomings coupled with existing active lead compounds, such as poor solubility, synthetic inaccessibility, high toxicity, acquired resistance, and metabolic instability. Moreover, scaffold hopping eliminates the need for repeated use of screening methods, which are often neither cost-effective nor efficient. Additionally, scaffold hopping offers measures to overcome the challenges coined with obtaining patent rights for the unmodified forms of natural products. It also enables expanding the intellectual properties (IP) space for companies.^16^ The term scaffold hopping was first used by Schneider in 1999 ^17^ and as a concept it can be understood as an extension of traditional replacement strategies such as isosterism defined by Langmuir, Grimm and Erlenmeyer in the early 1900s and further recruited bioisosterism explicated by Friedman in 1951 ^18^ and Thornber in 1979.^19^ The methods used for scaffold hopping are diverse, varying significantly in their level of sophistication. The simplest scaffold hops are often guided by hypotheses formulated by medicinal chemists.^15^ These modifications are not computer-aided and involve transpositions of a heteroatom within the heterocyclic backbone, adding or removing the heteroatom, and closing or opening the ring.^13^ Such modifications are usually sufficient to deduce the pharmacophore,^20^ but the molecular similarity, shape and electron distribution cannot be quantified.^21^ In contrast, *in silico* driven scaffold hopping leverages advanced modeling algorithms, such as shape matching, pharmacophore modeling, fragment replacement, and similarity searching, to explore chemical space more systematically.^13,14,22^ These *in silico* approaches rely on computational tools widely used in modern drug discovery, including virtual screening (VS), which prioritizes alternative scaffolds based on their predicted binding affinity to a biological target.^23,24^ Two major methods exist: (i) ligand-based VS (LBVS) and (ii) structure-based VS (SBVS). LBVS identifies candidate scaffolds with key similar chemical features critical for protein binding using molecular fingerprints and similarity assessment (e.g., Tanimoto score). SBVS uses 3D structural data from all sources, including X-ray crystallography, NMR spectroscopy, and the Protein Data Bank (PDB), to model receptor–ligand interactions. Molecular docking, the core technique of SBVS, predicts binding modes and estimates interactions’ strength between a small molecule—typically obtained from commercially available libraries (PubChem, ChEMBL, or ZINC etc.)—and protein target. SBVS, a validated tool for scaffold hopping applications, has received particular attention over the past decade due to significant advances in structural biology and genomics, which have facilitated a deeper understanding of the 3D structures of numerous validated biological targets.^23–27^ However, the use of in silico methods in scaffold hopping also has limitations; including scoring function accuracy, potential misinterpretation of alternative scaffolds, failure to reflection potential off-target interactions of new scaffold, and generally strong reliance on accurate input data.^28^ If scaffold hopping is driven computationally, the prerequisite for the success of the whole process relies heavily on the proper definition of the part of the molecule considered as a scaffold.^13^ The first globally accepted definition of molecular scaffold was introduced by Bemis and Murcko (BM scaffolds) in 1996.^29^ Despite its limitations,^30^ it has become a solid foundation for computational analysis. The BM scaffold is generated from the molecule by removing all the pendant substituents while retaining the aromatic systems and the linker connecting the aromatic systems. The HierS method (hierarchical scaffold clustering using topological chemical graphs), which builds on the algorithm for generating BM scaffolds, addresses certain ambiguities in extracting the molecular core. This method systematically organizes the related scaffolds into a unified network framework by breaking the original BM scaffold into all possible ring fragments.^31^ As an alternative approach to the HierS method, Scaffold Tree Algorithm systematically decomposes BM scaffolds. The Scaffold Tree Algorithm proposes possible structural variations of the scaffold within the form of tree diagrams.^32^

### Classification of Scaffold Hopping

From the historical point of view, scaffold hopping has been broadly understood in literature as a wide spectrum of modification, ranging from routine bioisosteric replacement to significant structural overhauls. However, the distinction between these approaches has often been blurred, with the definition of the process primarily left to the discretion of the medicinal chemist.^15^ This ambiguity was clarified by Sun and co-workers in 2012.^33^ This work emphasized that structural changes proposed as scaffold hopping are exclusively aimed at modifying the core of the molecule. Furthermore, the authors proposed categorizing the scaffold hopping into four degrees (1°–4°) based on the type of structural core change relative to the parent molecule. They also established a practical framework for classifying individual case studies across the field of drug development. Each degree of scaffold hopping, as defined by Sun and co-workers,^33^ is briefly discussed in the following sections. Examples are illustrated in Figure 1.

**Figure 1:**
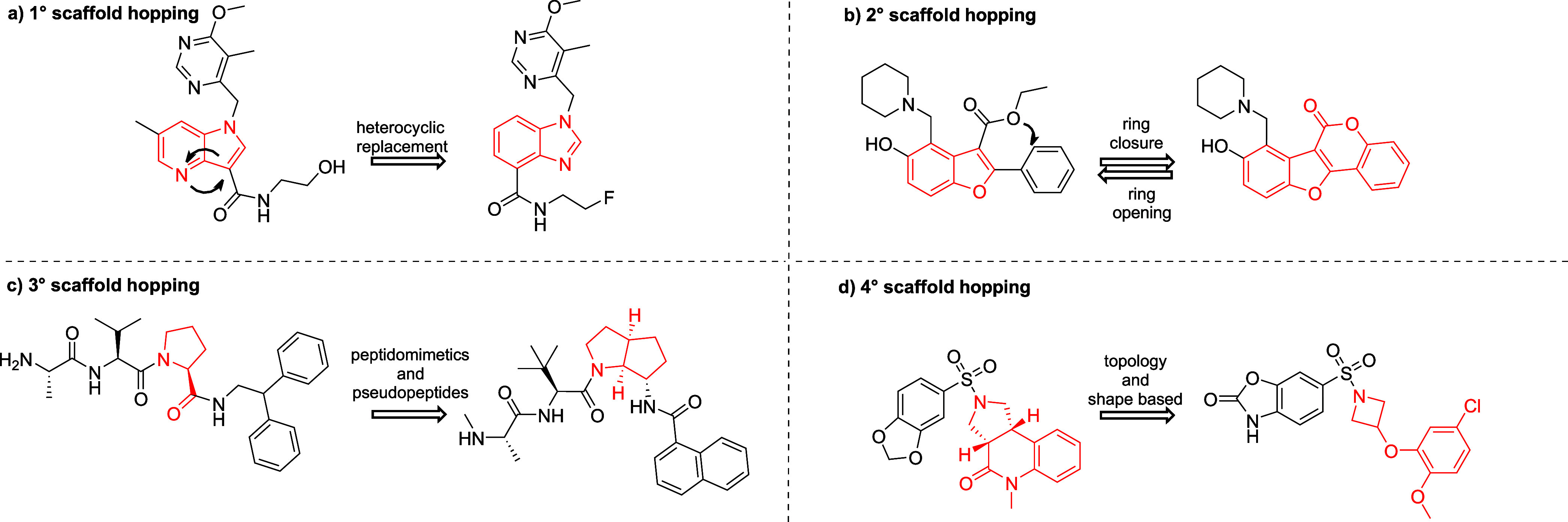
Four degrees of scaffold hopping with the representative as proposed by Sun and co-workers.^33^ (a) heterocyclic replacement. (b) Pseudoring structures: ring-opening and ring-closing. (c) Peptidomimetics and pseudopeptides. (d) Topology and shape-based overhauls.

Heterocyclic replacement (1° scaffold hopping, Figure 1a) represents the simplest form of scaffold hopping and is commonly employed in structure–activity relationship (SAR) studies with a relatively high success rate. This approach involves the substitution, addition, or removal of heteroatoms within the molecular backbone, as well as the replacement of one heterocycle with another of high similarity. It retains the spatial arrangement of the unaltered pharmacophore and adjacent groups, enabling the tuning of physicochemical properties, optimization of the pharmacokinetic (PK) profile, and identification of key ligand-target interactions.^15,16,33^ Although modifications to the parent scaffold are often minor and may lack significant novelty, these changes typically require a different synthetic approach.^33^ Therefore, according to Boehm et al.,^14^ these modifications can be considered novel. Evidence supporting this statement is provided by the strong structural similarity between the phosphodiesterase type 5 (PDE5) inhibitors sildenafil and vardenafil, which differ only in the position of a nitrogen atom yet are covered by separate patents.^34^ Nevertheless, such small structural modifications within the scaffold often result in limited changes to properties and provide minimal advantages in establishing a strong IP position.^33^

The ring opening and closure approach (2° scaffold hopping, Figure 1b) involves modulating the conformational flexibility of the molecular backbone, advancing toward more complex structural changes to create a new scaffold.^15^ The application of the ring closure approach enhances molecular rigidity, thereby reducing the entropic component of binding free energy, which often improves target engagement.^35^ Moreover, reducing the flexibility of the molecule can prevent undesirable off-target interaction.^16^ On the other hand, the lack of flexibility of the newly created scaffold can negatively impact the solubility and ADME properties.^36^ In contrast, the ring-opening approach is associated with increased molecular flexibility, allowing the ring-opened analogues to occupy dynamic binding pockets more effectively.^15^ Generally, 2° scaffold hopping is a valuable tool to control the number of free rotatable bonds or rings in the structure and boost the drug-like character of molecules.

3° scaffold hopping (Figure 1c), also referred to as peptidomimetics or pseudopeptides, is focused on the modifications to the primary structure of peptides and proteins. These structural changes involve the replacement of the amino acids with small structural motives that can maintain the spatial arrangement of the peptide chain, such as α-helices and β-sheets, ensuring its ability to interact with the secondary structure of the biological target.^15,33^ The hallmark of this approach lies in addressing the major limitations of natural peptides and proteins, including poor metabolic stability, rapid degradation by proteases, selectivity issues, and limited oral bioavailability.^15,16,33^

4° scaffold hopping (Figure 1d) encompasses the radical structural overhauls of the core in lead candidates and is almost exclusively driven by *in silico* methods.^15^ The application of this approach results in a completely new, unrelated scaffold that maintains only the crucial ligand-target interactions, electrostatic properties, and overall 3D shape of the molecule.^16^ While this approach appears highly promising, with the potential to overcome IP barriers and address resistance mechanisms to current therapeutics, it is rarely reported in the literature. This is likely because major structural changes often entail lower success rates, resulting in fewer case studies. Additionally, VS and 4° scaffold hopping are complementary strategies, often applied at different stages of the drug discovery process. While VS is typically employed for hit identification, scaffold hopping plays a key role in hit-to-lead optimization. Increasingly, these approaches are used complementarily with techniques such as docking guiding scaffold modification.^15,16,33^

As *in silico* methods are widely involved, particularly in the higher degrees of scaffold hopping, numerous software tools have been developed to facilitate the effective application of individual scaffold hopping strategies. For example, CAVEAT and Cresset Spark tools are widely used for identifying suitable scaffold replacements, leveraging conformational and electrostatic properties to guide the selection process.^33,37,38^ Scaffold hopping from natural products is often achieved via WHALES (Weighted Holistic Atom Localization and Entity Shape), the similarity-based method designed to generate novel synthetic mimetics.^39^ Despite the utilization of *in silico* methods in first degree of scaffold hopping is not always necessary, the MORPH method, with its library of drug-like scaffolds, can guide medicinal chemists toward more rational heterocyclic replacements and improved success rates.^33,40^

Although the aforementioned provide only a brief insight into scaffold hopping, it offers sufficient information necessary for further orientation in this perspective. It enhances understanding of the concept as it is applied across literature.

## The Aim and Scope of the Review

This perspective aims to analyze and discuss the applications of scaffold hopping in the discovery of new therapeutics for TB at various stages of development over the past two decades. Case studies included in this perspective were selected not only based on the clinical relevance of the parent molecules and their scaffold-derived analogs, but also on the availability of comprehensive data and literature that capture the full development context of the respective compound classes. The chosen families of compounds allow unambiguous scaffold identification within molecules, following the scaffold definition algorithms proposed by Bemis and Murcko.^29^ While drug discovery efforts in the field of TB encompass a vast number of studies involving scaffold hopping, it is virtually impossible to include all of them within the scope of this perspective. Nevertheless, the chosen case studies represent relevant compound classes and provide a clear rationale for the application of scaffold hopping in the context of drug design. For individual scaffold hops, emphasis will be placed on the modifications and structural considerations within the core of lead compounds that result in desired changes in properties such as increased efficacy, reduced toxicity, or improved solubility, while also addressing the associated limitations and challenges encountered during the drug discovery process. For improved comparability and evaluation of activity parameters (e.g., MIC, IC_50_), cytotoxicity (e.g., IC_50_) and other assessed attributes of parent molecules and novel derivatives within individual case studies, certain original values have been recalculated and standardized to a uniform unit, molar concentration [M]. Where applicable, the impact of structural modifications resulting in the formation of new chemical entities will be evaluated in terms of their binding interactions within the ligand–protein complex, accompanied by a comparative analysis to the parent molecule. To facilitate navigation in the text, individual case studies highlighting the recent advances of scaffold hopping in TB drug development to date are categorized according to the classification framework proposed by Sun and co-workers.^33^

### Application of Scaffold Hopping in TB Drug Discovery

#### 1° Scaffold Hopping

Given that a high proportion of drugs contain at least one heterocycle, first-degree scaffold hopping is widely utilized and frequently considered for establishing SAR, increasing synthetic accessibility, addressing key deficiencies of lead molecules during preclinical development, and expanding IP space.^15^

Targeting energy metabolism in *Mtb* is a promising strategy for developing new TB drugs. Cytochrome *bcc*, a critical component of the mycobacterial respiratory system, plays an essential role in the electron transport chain (ETC) and ATP synthesis. Inhibiting cytochrome *bcc* disrupts the proton motive force (PMF), halting ATP production.^41^ Notably, the QcrB subunit of cytochrome *bcc* has emerged as a pivotal target for anti-TB drugs, with the most advanced inhibitor, Q203 (telacebec; Figure 2), currently undergoing phase II of clinical trials.

**Figure 2:**
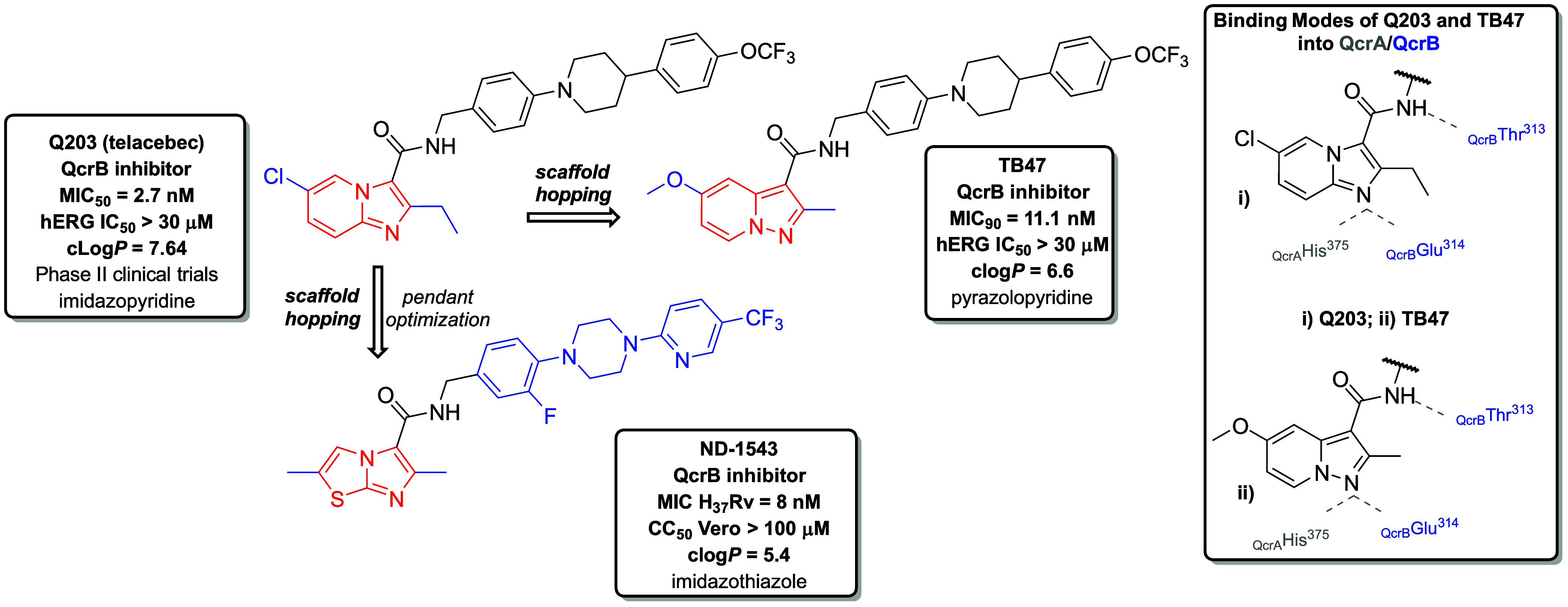
Illustration of 1° scaffold hopping applied to the clinical candidate Q203, an inhibitor of the QcrB subunit, as utilized by Tang and co-workers,^45^ leading to the identification of the novel preclinical candidate TB47. The pyrazolopyridine scaffold of TB47 preserved a key hydrogen bond with Glu314 of the QcrB subunit. TB47 also revealed an additional hydrogen bond with His375 of the QcrA subunit within the cytochrome *bcc* complex, enhancing its binding interactions. ND-1543 is also displayed as another successful example of first-degree scaffold hopping applied to Q203, albeit less favorable compared to parent Q203.

Q203, a member of the imidazopyridine class discovered by Pethe et al., exhibits potent antimycobacterial activity (*Mtb* H_37_Rv MIC_50_ = 2.7 nM after 21 days of incubation), low hERG (the human ether-*a-go-go* related gene) inhibition (hERG IC_50_ > 30 μM), and favorable pharmacokinetic (PK) properties.^42,43^ As Q203 selectively inhibits cytochrome *bcc*, it acts as a bacteriostatic agent.^44^ Tang et al. applied a scaffold hopping approach with Q203, modifying the imidazopyridine core by transferring the nitrogen from the position 4 to create a novel pyrazolopyridine class of compounds, which led to the identification of the lead compound TB47 (Figure 2). The newly synthesized pyrazolopyridine scaffold in TB47 retains a similar 3D conformation and electronic properties to the original structure, maintaining the critical H-bond with Glu314 residue of QcrB.^45,46^ TB47 demonstrated sustained efficacy against DR-TB strains and exhibited comparable activity against *Mtb* as Q203 (MIC_90_ H_37_Rv = 11.1 nM after 7 days), low cardiotoxicity (hERG IC_50_ > 30 μM), a 10-fold lower clog*P* value compared to Q203 and good oral bioavailability in mice models.^45,47^

In 2016, Moralski et al. also employed scaffold hopping with Q203 as a part of their SAR study to generate a novel lead compound ND-1543 (Figure 2), bearing imidazo­[2,1-*b*]­thiazole scaffold while retaining its inhibitory activity against QcrB. ND-1543 revealed comparable activity against replicating *Mtb* (MIC H_37_Rv = 8 nM) as Q203, exhibited no cytotoxicity at the maximum tested concentration (CC_50_ VERO cells >100 μM), did not inhibit the major CYP enzymes (IC_50_ values > 10 μM), and showed moderate stability in human liver microsomes (HLMs) (*T*_1/2_ = 28 min). However, ND-1543 exhibited relatively poor PK profile *in vivo* (BALB/c mice) and also poor efficacy in a chronic TB-infected BALB/c mouse model, as indicated by a 0.3 log_10_ CFU reduction in bacterial lung burden after 30 days of oral dosing at 200 mg/kg, relative to the untreated control.^48,49^ Obviously, this simple and well-proven approach can also lead to less favorable compounds.

*Mtb* possesses a unique cell wall structure composed of a thick peptidoglycan layer linked to arabinogalactan and an outer membrane enriched with mycolic acids. Additionally, the cell wall includes essential components such as lipoarabinomannan and lipomannan. The impermeability and structural robustness of the cell wall confer *Mtb* a unique, innate, and nonspecific resistance to antimicrobial agents, posing a significant challenge in developing new anti-TB drugs. On the other hand, the *Mtb* cell wall contains numerous enzymes and transporters that serve as promising unique targets for therapeutic intervention. Therefore, designing agents that disrupt mycobacterial cell wall biosynthesis represents a highly effective strategy for TB treatment.^50^

Decaprenylphosphoryl-β-d-ribose 2́-epimerase (DprE1; EC 1.1.98.3) is a part of crucial enzymatic complex that catalyzes the epimerization of decaprenylphosphoryl-β-d-ribose (DPR) to decaprenylphosphoryl-β-d-arabinose (DPA), an essential step in the biosynthetic pathway generating arabinogalactan and lipoarabinomannan.^51^ Inhibition of this target represents a promising strategy to disrupt cell wall synthesis. Scientific efforts have led to the development of four clinical candidates with a mechanism of action (MoA) centered on DprE1 inhibition. These include the noncovalent inhibitors 1,4-azaindole TBA-7371 (phase II) and 3,4-dihydrocarbostyril derivative OPC-167832 (quabodepistat, phase II), and covalent inhibitors from benzothiazinone family such as BTZ-043 (phase II) and PBTZ-169 (macozinone, phase II).^52^

TBA-7371 (Figure 3a), a 1,4-azaindole derivative discovered by researchers at AstraZeneca, was identified as a noncovalent inhibitor of DprE1 through mass spectrometric analysis.^52,53^ TBA-7371 revealed not only potent enzymatic inhibition of DprE1 (IC_50_ DprE1 = 10 nM), but also good whole-cell activity (MIC H_37_Rv = 0.78 μM). However, subsequent investigation showed that this derivative also inhibits phosphodiesterase 6 (PDE6 IC_50_ = 4 μM), raising concerns about potential ocular side effects.^54^ Subsequently, the same research group employed scaffold hopping with TBA-7371, relocating the N4 nitrogen within the azaindole core to the N3 position and shifting the amide side chain from the C3 position to the C4 position. This structural modification resulted in the compound **1** (Figure 3a) featuring a benzimidazole (BI) scaffold.^55^ The overlay of TBA-7371 and the BI analogue **1** showed that the new derivative **1** retains a binding mode similar to TBA-7371. The interaction mechanism of TBA-7371 within the DprE1 active site was proposed through docking studies utilizing the crystal structure of DprE1 complexed with TCA1 (PDB ID: 4KW5). This predicted binding interaction is characterized by CH-π contact between the pyridine ring of the azaindole and Tyr314, and a bifurcated hydrogen bond involving the N4 atom, the carbonyl oxygen of the amide side chain, and Ser228. Additionally, the amide NH forms hydrogen bond contact with the redox cofactor, flavin adenine dinucleotide (FAD), while the terminal OH group is positioned within the hydrogen-bonding distance of Asn385. Transposing the amide side chain from the C4 to C3 position in **1** maintains sufficient proximity to form the key hydrogen bond between the carbonyl oxygen in **1** and Ser228, and the unsaturated six-membered ring of BI in **1** preserves the CH–π interaction with Tyr314. The similar spatial arrangement of the new BI analogue **1** retains the original hydrogen bond between the amide NH group and FAD, as well as the close proximity of terminal fluorine atom in the BI derivative **1** to Asn385.^54,55^ Biological evaluation of **1** showed MIC value 2-fold higher than that of TBA-7371 (MIC H_37_Rv = 1.56 μM). The biological target of **1** was indirectly determined by testing the **1** against 1,4-azaindole-resistant strains (DprE1 with the Y314H mutation). A significant shift in MIC was observed compared to wild-type *Mtb* (MIC H_37_Rv = 1.56 μM vs MIC DprE1-Y314H = 25 μM), suggesting that the **1** shares the same MoA as TBA-7371. Compound **1** was also evaluated *in vivo* in the BALB/c mouse model of chronic TB infection. The efficacy of **1** was demonstrated by a reduction in bacterial burden of 1.5 log_10_ CFU in the lungs and 1.0 log_10_ CFU in the spleen after 4 weeks of treatment at a dose of 30 mg/kg. Notably, **1** revealed limited CYP and hERG inhibition (CYPs IC_50_ values > 50 μM (21A2, 2C9, 2C19, 2D6, 3A4); hERG IC_50_ > 33 μM), and excellent oral bioavailability in rats (*F*_oral_ = 114%).^48,55^

**Figure 3:**
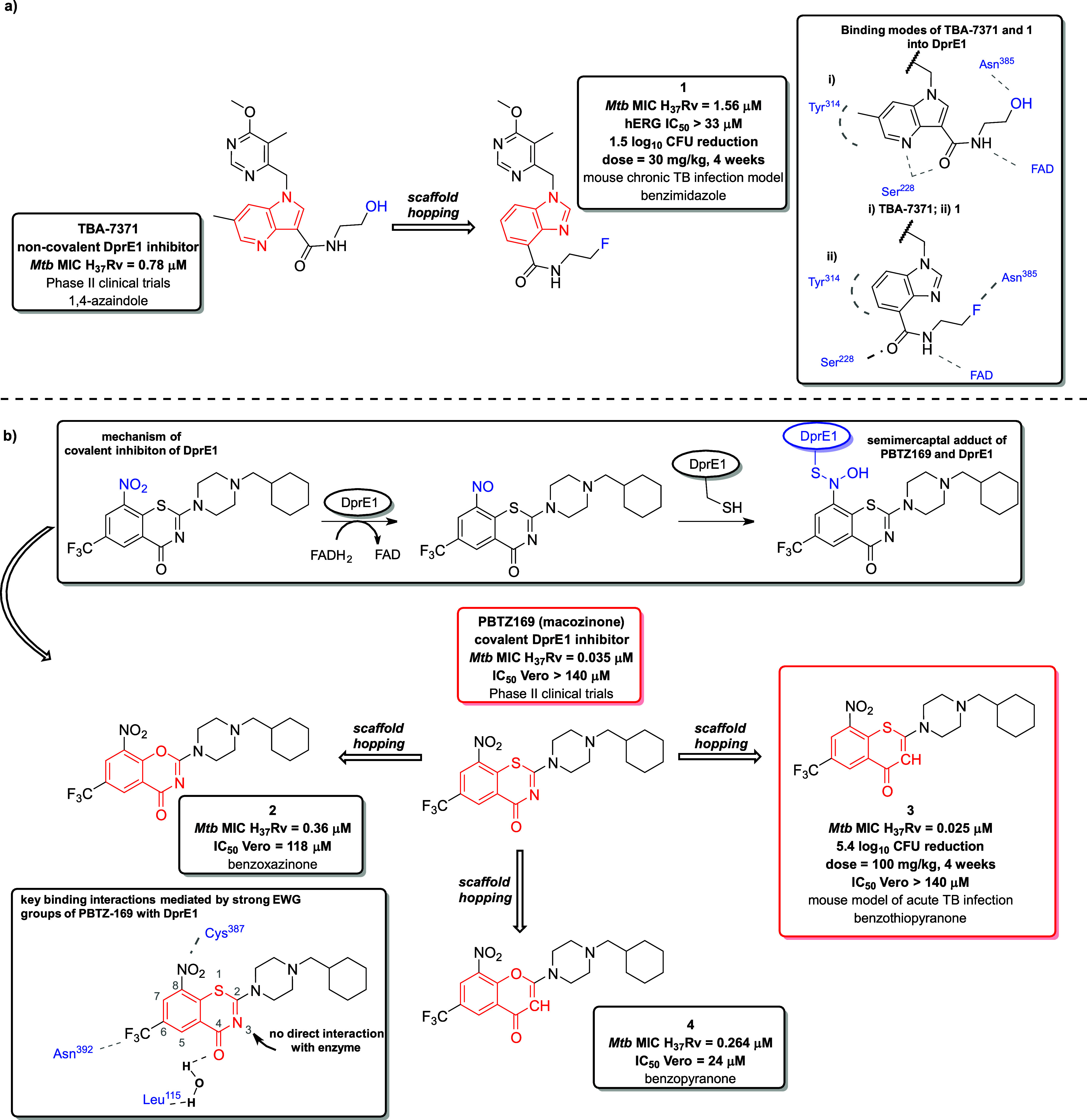
(a) 1° degree scaffold hopping applied to the clinical candidate TBA-7371, resulting in a new derivative **1** bearing a benzimidazole scaffold, which demonstrated *in vivo* efficacy in a murine model of chronic TB infection. Key hydrogen bonds between the active site of DprE1 and TBA-7371, and **1** are illustrated with gray, straight dashed lines. The CH–π interaction between the heterocyclic core of TBA-7371 and **1**, and Tyr314 is displayed in a dashed circular line. (b) 1° scaffold hopping performed on the clinical candidate PBTZ-169 leading to the discovery of PBTZ-169 analogs harboring benzoxazinone (**2**), benzothiopyranone (**3**) and benzopyranone (**4**) scaffolds. Key interactions between the electron-withdrawing groups (EWG) attached to the central core of PBTZ-169 and the active site of DprE1 are depicted by gray dashed lines.

The BTZs class was first discovered in 2009 by V. Makarov and S. T. Cole.^56^ Their efforts led to the development of two clinical candidates, BTZ-043 and its related compound, PBTZ-169 (macozinone) (Figure 3b).^56,57^ These compounds target cell wall synthesis by inhibiting DprE1, serving as prodrugs that require in situ reduction^58^ to generate reactive nitroso derivatives from its respective nitro group. These derivatives form a covalent bond with the DprE1 enzyme by creating a semimercaptal adduct through a reaction with the thiol group of the cysteine from residue Cys387 in DprE1, leading to the enzyme’s irreversible inactivation (Figure 3b). Thus, this class represents covalent DprE1 inhibitors. BTZ-043 is a highly potent *in vitro* inhibitor of DprE1, demonstrating consistent activity against both *Mtb* H_37_R_v_, MDR- and XDR-TB strains (MIC = 2.3 nM). BTZ-043 is currently undergoing phase II of clinical trials.^52^ However, the exceptionally low MIC value of BTZ-043 and it is *in vivo* efficacy were not sustained, and several drawbacks of the compound emerged, such as the presence of a chiral center and relatively high synthesis costs. All these aspects led the researchers to investigate modifications at position 2 of BTZ-043 further. These efforts resulted in the discovery of the covalent DprE1 inhibitor 2-piperazino-benzothiazinone, PBTZ-169 (macozinone), which is currently in phase II of clinical trials.^52,56,57,59^ PBTZ-169, which lacks a chiral center and has lower synthetic costs, exhibited improved cytotoxicity profile (PBTZ-169 TD_50_ HepG2 = 127 μM vs BTZ-043 TD_50_ HepG2 = 12 μM), greater potency (*Mtb* H_37_Rv MIC_99_ = 0.65 nM), and significantly improved efficacy at lower concentrations in a murine model of chronic TB compared to BTZ-043.^57^ The cocrystal structure of PBTZ-169 with DprE1 (PDB ID: 4NCR) reveals that the essential fragments of the molecule to provide key interactions within the DprE1 active site involve the sulfur atom, a carbonyl group within the thiazinone ring, a strong electron-withdrawing CF_3_ group at position 6, and an indispensable nitro group at position 8. However, the nitrogen atom at position 3 does not participate in direct interactions with the enzyme.^57^ Building on this observation, Peng et al. employed scaffold hopping by substituting the nitrogen at position 3 with a bioisosteric carbon atom and replacing the sulfur atom with a bioisosteric oxygen. These modifications led to the development of three structural analogues of PBTZ-169, bearing benzoxazine (compound **2**), benzothiopyranone (compound **3**), and benzopyranone (compound **4**) scaffolds, while preserving the original positions of the CF_3_ and nitro groups (Figure 3b). Compared to PBTZ-169, the benzoxazine derivative **2** displayed 1 order of magnitude reduction in activity (compound **2** *Mtb* MIC H_37_Rv = 0.36 μM vs PBTZ-169 *Mtb* MIC H_37_Rv < 0.035 μM, respectively) and a higher *in vitro* cytotoxicity (compound **2** IC_50_ Vero = 118 μM vs PBTZ-169 IC_50_ Vero >140 μM, respectively). Likewise, replacing the benzothiazinone in PBTZ-169 with a benzopyranone bearing analogue **4** caused a significant increase in cytotoxicity (compound **4** IC_50_ Vero = 24 μM vs PBTZ-169 IC_50_ Vero >140 μM) and 8-fold decrease in activity (compound **4** *Mtb* MIC H_37_Rv = 0.264 μM vs PBTZ-169 *Mtb* MIC H_37_Rv < 0.035 μM). This suggests that the sulfur atom is essential to maintain the favorable *in vitro* safety and activity of the BTZ class. In contrast, replacing the benzothiazinone scaffold in PBTZ-169 with benzothiopyranone **3** is a successful example of scaffold hopping demonstrated in the work of Peng et al. Indeed, **3** exhibited the highest activity among the three derivatives of PBTZ-169 (*Mtb* MIC H_37_Rv < 0.025 μM) while also demonstrating the lowest cytotoxicity (IC_50_ Vero >140 μM). Additionally, *in vivo* evaluation of **3** using a BALB/c mouse model of acute TB infection revealed a significant reduction of 5.4 log_10_ CFU in the lungs after 3 weeks of treatment at a dose of 100 mg/kg. However, like PBTZ-169, derivative **3** exhibited very low bioavailability (*F*_oral_ = 13%). Favorable safety data of **3** indicated a low risk of cardiotoxicity (hERG IC_50_ > 30 μM) and limited CYP enzyme inhibition (CYPs IC_50_ > 50 μM; 1A2, 2C9, 2C19, 2D6, 3A4).^60^

Mycobacterial membrane protein large 3 (MmpL3), a member of the Resistance, Nodulation, and Division (RND) protein superfamily, plays a vital role in several cellular processes, including energy metabolism and cell homeostasis. MmpL3 also participates in mycobacterial cell wall synthesis, where it functions as a transporter. This RND transporter uses PMF to transport trehalose monomycolates (TMM) from the cytoplasm. TMM subsequently acts a substrate for mycotransferases, enzymes crucial for the biosynthesis of mycomembrane components, which are essential for maintaining the structural integrity of the mycobacterial cell wall. The inhibition of MmpL3 leads to the death of *Mtb*, making it a suitable target for potential anti-TB agents.^61^ The family of MmpL3 inhibitors encompasses a diverse range of compounds, including carboxamide derivatives (NITD-304 and NITD-349), adamantyl ureas (AU1235), pyrroles (BM212, BM635), and benzimidazoles (C215).^62^ Notably, the ethylenediamine derivative SQ109, the most advanced MmpL3 inhibitor to date, is currently undergoing phase II clinical trials.^62^

The first MmpL3 inhibitor from the class of 1,5-diphenylpyrroles, compound BM212 (Figure 4), was identified through random screening of a library of azole compounds. BM212 demonstrated moderate activity against replicating *Mtb* strains (MIC H_37_Rv = 5 μM).^63^ Similar to SQ109, C215 and other MmpL3 inhibitors, BM212 also exhibits a PMF uncoupling effect and additionally shows activity against nonreplicating *Mtb* strains grown under low oxygen conditions (MIC *Mtb* H_37_Rv = 18.5 μM; LORA assay).^61^ A subsequent SAR study resulted in the development of compound BM635 (Figure 4), displaying submicromolar activity (*Mtb* MIC H_37_Rv = 0.12 μM) and an acceptable safety profile (CC_50_ HepG2 = 40 μM; hERG IC_50_ = 10 μM). BM635 also demonstrated efficacy in a murine model of acute TB infection with 2.0 log_10_ CFU reduction in lungs after 8 days of treatment at 50 mg/kg. However, BM635 exhibited very low kinetic solubility (CLND solubility <1 μM), limiting its potential for further development.^64^ To overcome this limitation, the structure of BM635 was modified by altering the substitution at the N1 position within the central pyrrole ring. Replacing the original 4-fluorophenyl group at N1 position of BM635 with isopropyl moiety resulted in the development of compound **5** (Figure 4). Compound **5** exhibited comparable *in vitro* activity (*Mtb* MIC H_37_Rv = 0.15 μM) and *in vivo* efficacy (1.5 log_10_ CFU reduction in lungs after 8 days of treatment at 50 mg/kg in an acute TB infection mouse model) to BM635. Additionally, **5** maintained a similar safety profile (CC_50_ HepG2 = 20 μM; hERG IC_50_ = 16 μM) and demonstrated excellent kinetic solubility (chemiluminescent nitrogen detection, CLND, solubility = 199 μM). Nevertheless, **5** had a poor oral bioavailability (*F*_oral_ = 1% in C57BL mice).^65^ To further enhance the 1,5-diphenyl pyrrole class, scaffold hopping was employed to replace the original pyrrole core of BM635 with a pyrazole scaffold, yielding compound **6** (Figure 4). Compound **6** demonstrated 2-fold lower activity (*Mtb* MIC H_37_Rv = 0.30 μM) compared to BM635, low cytotoxicity (CC_50_ HepG2 = 32 μM), and good aqueous solubility (CLND solubility = 152 μM). However, a higher potassium channel affinity of **6** (hERG IC_50_ = 6.3 μM) suggests a potential cardiotoxicity risk. In a murine model of acute TB infection, the pyrazole derivative **6** achieved 1.5 log_10_ CFU reduction in lung bacterial burden after 4 days of treatment at a dose of 200 mg/kg.^66^ As the binding mode of the pyrazole derivative **6** has not yet been elucidated, its potential interaction with MmpL3 can only be hypothesized. Compound **6** exhibits structural similarity and shares a heterocyclic scaffold with the CB1 receptor antagonist rimonabant, demonstrating weak anti-TB activity. Zhang et al. performed a microscale thermophoresis assay, determining that rimonabant fits well to the same binding pocket of SQ109 and AU1235, and interacts with MmpL3 with a *K*_d_ value of 29 μM. From a complex of *Mycobacterium smegmatis* MmpL3 and rimonabant (PDB ID: 6AJI), it is evident that the heterocyclic pyrazole core is involved in hydrophobic interactions with the Gly641, Leu642, and IIe253 residues. A complementary docking study of BM212 and MmpL3 suggests that BM212 and rimonabant are accommodated in the same hydrophobic pocket with their heterocyclic core, forming contact with IIe253 residue.^67^ Thus, the structural similarity between compound **6**, rimonabant, and BM212, along with their good anti-TB activities, suggest that **6** likely shares a similar binding mode to BM212 and rimonabant.

**Figure 4:**
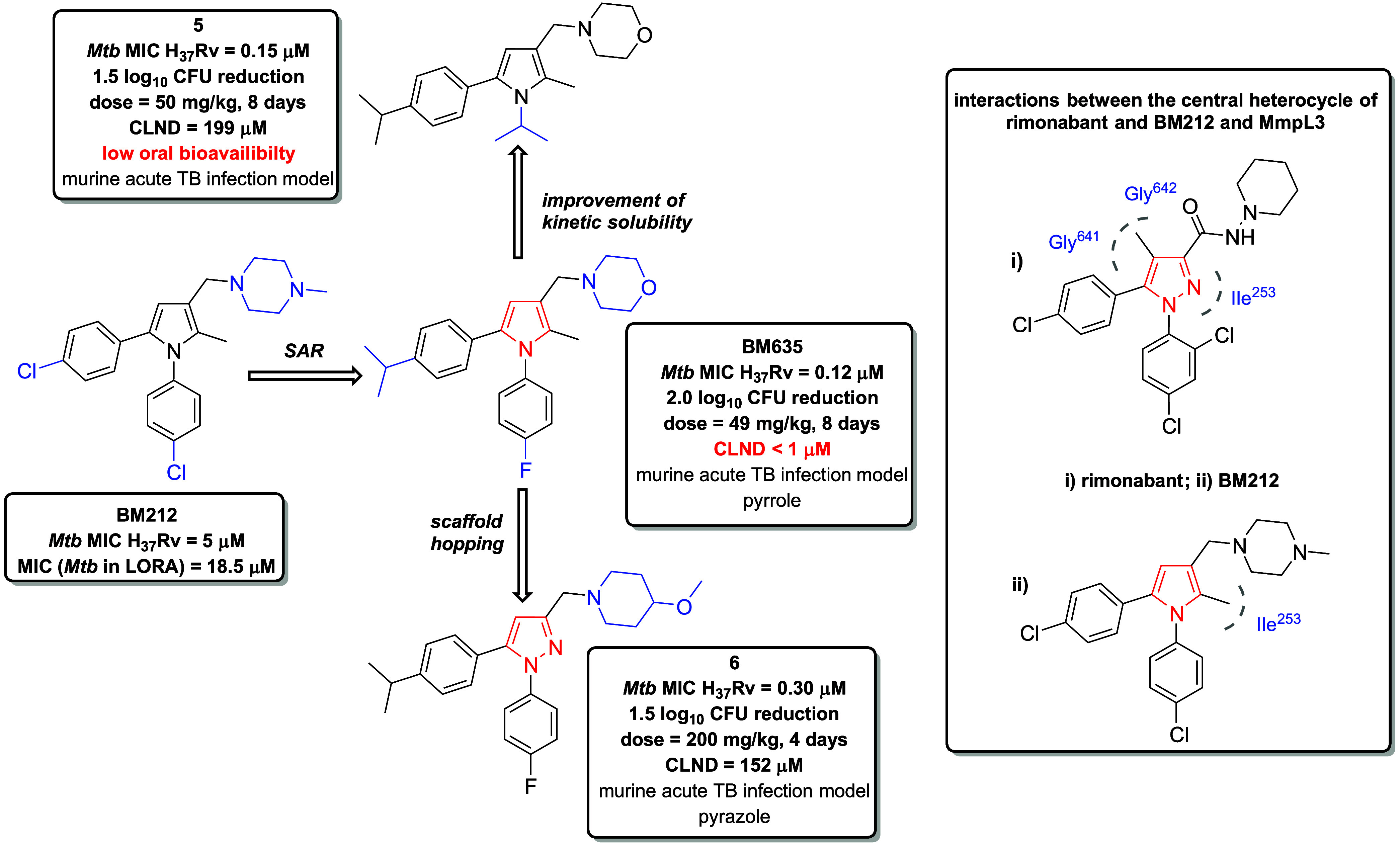
Development pipeline of the previously discovered 1,5-diphenylpyrrole derivative BM212, inhibiting MmpL3. Structural modifications (highlighted in blue) across this class of compounds were undertaken to improve unfavorable physicochemical properties (e.g., BM635) and the PK profile of **5**. 1° Scaffold hopping (shown in red), replacing the pyrrole core in BM212 and BM635 with a pyrazole moiety in compound **6**, was implemented as a terminal step in the optimization of these MmpL3 inhibitors to enhance the overall drug-like characteristics across this class of derivatives. For illustration, the binding mode of BM212 highlights key interactions between the heterocyclic backbone and the active site of MmpL3. These hydrophobic interactions (displayed in gray dashed circular lines) are provided for BM212 and the structurally related rimonabant (PDB ID: 6AJI; MmpL3 of *M. smegmatis* complexed with rimonabant). For comparative analysis of BM212, rimonabant and compound **6**, it should be noted that the binding mode of pyrazole **6** has not been described in the literature; hence, this scheme serves only as a representation of its putative binding mode.

#### 2° Scaffold Hopping

The second-degree of scaffold hopping accounts for structural modifications that result in the formation of a new heterocyclic core through ring closure or, alternatively, the elimination of the original heterocyclic core via ring opening. This approach impacts molecular conformation by modulating the rigidity or flexibility of the entire molecule. These structural modifications can substantially influence enzyme-ligand interactions, making it challenging to predict their effects without comprehensive structural data on the biological target. The following sections will outline specific applications of second-degree scaffold hopping in the context of TB drug discovery.

#### Ring Closure

Protein synthesis is crucial for the survival and replication of *Mtb*, consuming approximately 50% of the total energy required for bacterial growth. The terminal step in protein synthesis, mRNA translation, takes place on the bacterial 70S ribosome, composed of two subunits (30S and 50S). Differences in the translation machinery between species facilitate the design of drugs that selectively target *Mtb*.^68^

Structural studies have shown that oxazolidinone antibiotics specifically bind to the A-site of the bacterial ribosome.^69^ Nevertheless, LNZ (Figure 5), the first oxazolidinone-based antibiotic approved for treating DR-TB, has been associated with toxicity. This is primarily attributed to its inhibition of mitochondrial protein synthesis (MPS) and monoamine oxidases, resulting in serious adverse effects such as myelosuppression and peripheral neuropathy, and unwanted serious drug interactions.^70^ To overcome these limitations, extensive research has focused on developing oxazolidinone derivatives over the past two decades. Among these, three compounds, namely sutezolid, delpazolid, and TBI-223, have shown improved safety profiles and are currently undergoing clinical trials for TB treatment.^52^ Sutezolid (Figure 5) is a thiomorpholine analogue of LNZ discovered in the 1990s. It has shown superior *in vitro* and *in vivo* efficacy against *Mtb* compared to LNZ and has advanced to phase II clinical trials.^48,52,69^ In 2020, Zhao et al. employed scaffold hopping to optimize the lead compound, resulting in the discovery of a conformationally constrained analogue of sutezolid, designated as OTB-658 (Figure 5). OTB-658 surpassed the efficacy of sutezolid against *Mtb* (OTB-658 MIC_90_ *Mtb* H_37_Rv = 0.08 μM vs sutezolid MIC_90_ *Mtb* H_37_Rv = 0.28 μM, respectively), and demonstrated a favorable safety profile, showing no cytotoxicity at the highest tested concentration (CC_50_ > 168 μM Vero and HepG2 cell lines). Among all possible configurations of the new benzoxazinyl-oxazolidinone scaffold in OTB-658, the 3S, 3aS diastereomer was identified as critical for antimycobacterial activity, as validated by X-ray analysis of the core structure.^71^ This derivative also showed significantly reduced inhibition of MPS compared to sutezolid and LNZ (IC_50_ > 100 μM, vs 8.2 μM and 8.0 μM, respectively), as well as reduced inhibition of monoamine oxidases A and B isoforms (OTB-658 MAO-A IC_50_ > 45 μM; MAO-B IC_50_ = 3.2 μM vs sutezolid MAO-A IC_50_ = 13 μM, MAO-B IC_50_ = 0.7 μM). Furthermore, OTB-658 demonstrated superior efficacy in a BALB/c mouse model of acute TB infection compared to LNZ, achieving a 5.1 log_10_ CFU reduction at 100 mg/kg, whereas LNZ yielded a 3.0 log_10_ CFU reduction at the same dose after 3 weeks of treatment. These excellent *in vivo* results prompted further testing of OTB-658 and LNZ in a BALB/c mouse model of chronic TB infection, where OTB-658 exhibited higher efficacy after 8 weeks at 25 mg/kg compared to LNZ applied at the same dose. Since OTB-658, like sutezolid, contains a thiomorpholine group, *in vivo* metabolic oxidation to a less active sulfoxide metabolite was observed. Nevertheless, its favorable *in vivo* PK profile, low hERG channel affinity (IC_50_ > 30 μM), and minimal inhibition of CYP enzymes (IC_50_ values > 45 μM across a panel of five isoforms) allowed this derivative to advance to preclinical development, aiming to evaluate the potential replacement of LNZ with OTB-658 in anti-TB regimens.^71^

**Figure 5:**
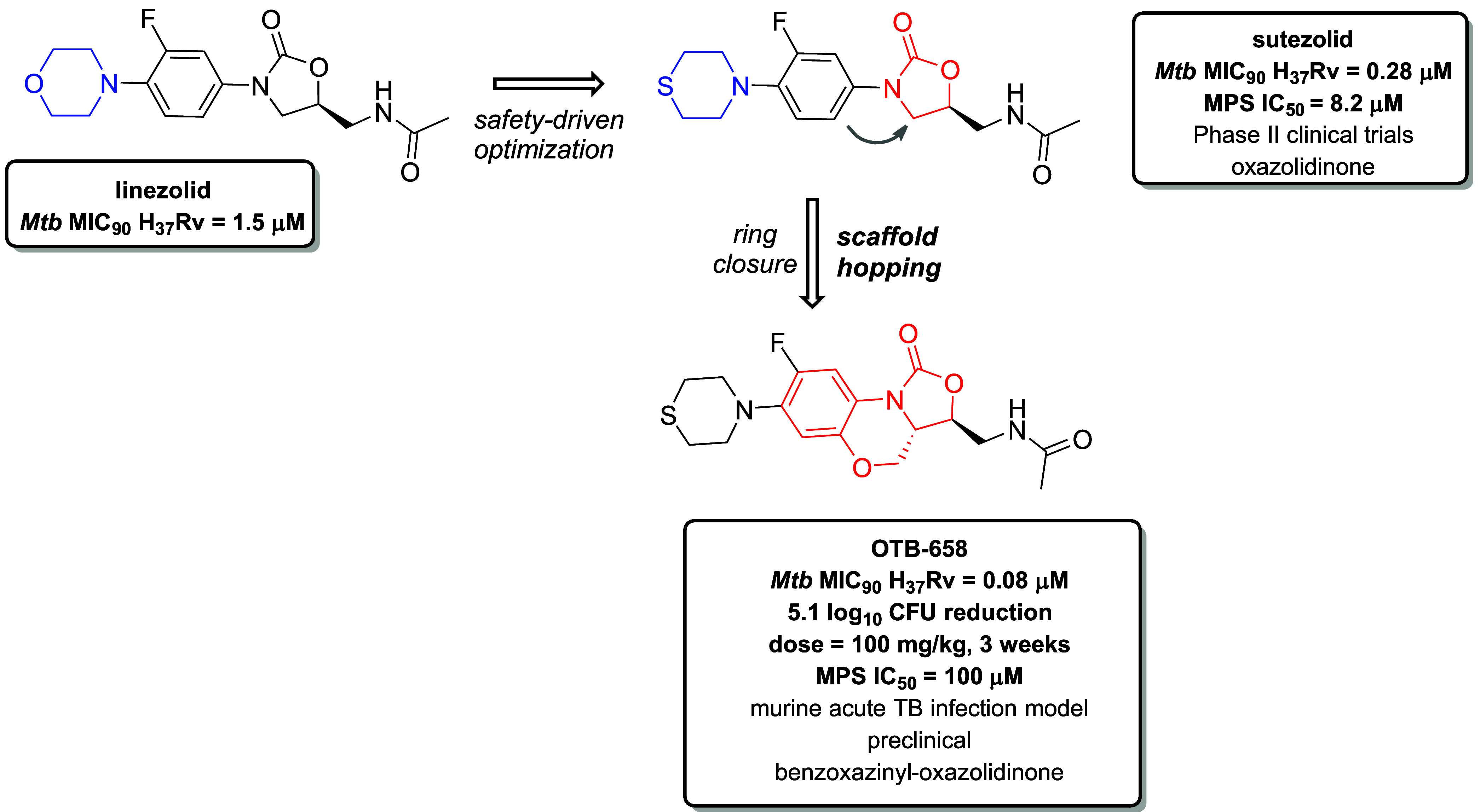
Application of 2° scaffold hopping in the class of oxazolidinone antibiotics (ATBs), used for the treatment of drug-resistant TB. Cyclization of the oxazolidinone core and the adjacent *N*-phenyl ring in sutezolid yielded a novel tricyclic benzoxazinyl-oxazolidinone scaffold in OTB-658. This modification effectively mitigated the major limitation of the oxazolidinone ATBs, namely the inhibition of MPS, which leads to severe side effects. The excellent *in vitro* and *in vivo* efficacy of OTB-658, along with its generally favorable safety profile, allowed the OTB-658 to advance to preclinical studies, aimed at evaluating its potential to replace LNZ in TB treatment regiments.

Polyketide synthases (PKS; EC 2.3.1.-) have emerged as a promising druggable target in TB treatment. These enzymes are involved in the biosynthesis of mycolic acids in mycobacteria, and their function is crucial for maintaining the integrity of the mycobacterial cell wall, which contributes to the intrinsic resistance of these pathogens to many antimicrobial agents. Despite their importance, they remain a relatively under-investigated group of enzymes. In *Mtb*, PKS-13, the member type-I PKS family, catalyzes the final step in the biosynthetic pathway leading to mycolic acids. The process involves a Claisen-type condensation between the C_26_ α-alkyl branch (from FAS-I biosynthesis) and the C_40–60_ meromycolate precursor (from FAS-II biosynthesis), forming the mycolic acids building blocks, which are subsequently translocated into the periplasm (Figure 6c).^72,73^

**Figure 6:**
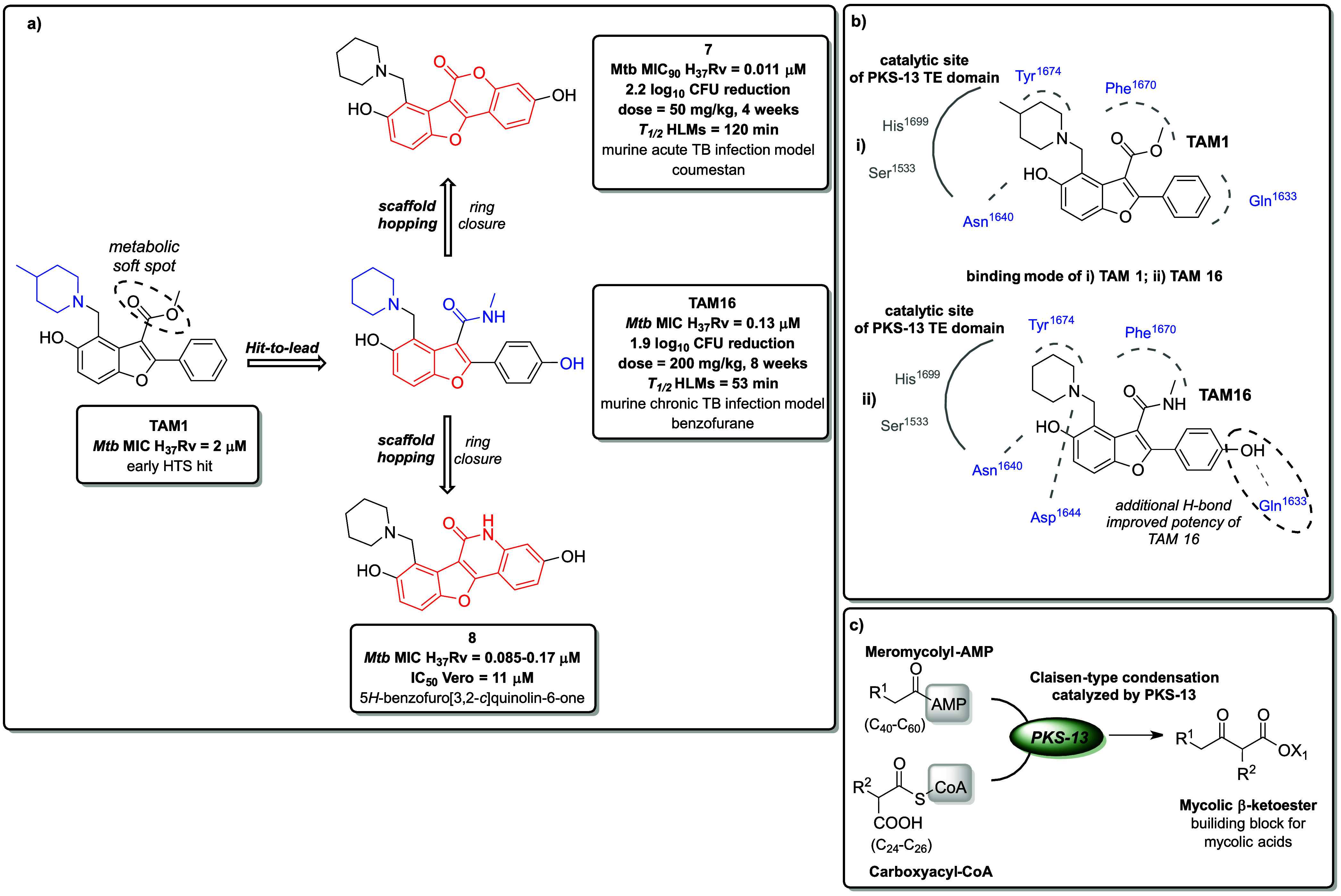
(a) Metabolism-driven 2° scaffold hopping applied to a novel class of TE-PKS13 inhibitors leading to the coumestan derivative **7** with efficacy in a murine model of acute TB infection and enhanced microsomal stability in HLMs compared to the hit compound TAM1 and lead compound TAM16. Subsequent intramolecular lactamization of TAM16 led to the discovery of derivative **8**, bearing 5H-benzofuro­[3,2-*c*]­quinolin-6-one scaffold, which demonstrated promising *in vitro* activity against *Mtb* H_37_Rv. (b) Docking of **7** within the comparative analysis of its binding mode with the parent molecule TAM16 revealed that the top-scored pose of **7** aligns with the previously identified binding mode of TAM16 (PDB ID: 5V3Y). The binding modes of TAM1 and TAM16 are illustrated, with critical H-bonds are represented as gray dashed straight lines and other binding interactions are depicted as gray dashed circular lines. Proximal residues in the catalytic site of PKS-13 TE domain are highlighted in gray. (c) PKS13 catalyzes the final condensation step in the biosynthesis of mycolic acids.

PKS-13 consists of five domains: two acyl carrier protein (ACP) domains, a β-ketoacyl domain, an acyltransferase domain, and a C-terminal thioesterase (TE) domain. In 2013, the Sachettini group identified the benzofuran derivative TAM1 ^74^ (Figure 6a) through high-throughput screening (HTS), which showed activity against *Mtb* H_37_Rv (MIC = 2.0 μM). Whole-genome sequencing and recombineering of resistance mutations revealed that TAM1 inhibits PKS-13. Furthermore, TAM1-resistant strains of *Mtb* harbored two mutations (D1644G and D1607N) in the TE domain of PKS-13, indicating that TAM1 binds to the TE domain. Such finding was further confirmed by X-ray cocrystal structure analysis (PDB ID: 5V3X).^72,74^ However, the ester bond in TAM1 is prone to hydrolysis, leading to the formation of an inactive carboxylic acid metabolite. Additionally, hydroxylation at the phenolic region of the molecule upon incubation with mouse liver microsomes (MLMs) is tolerated without a significant loss of activity. Several studies have focused on enhancing the metabolic stability of TAM1. The Sachettini group reported enhanced metabolic stability through the bioisosteric replacement of the ester bond with an amide bond, along with the introduction of a hydroxyl group at the 4-position of the phenyl ring. These modifications led to the discovery of the metabolically more stable compound TAM16 (Figure 6a), active against both DS-TB (*Mtb* MIC_90_ H_37_Rv = 0.13 μM) and MDR/XDR clinical isolates (MIC ranges between 0.05–0.25 μM). TAM16 also demonstrated efficacy in a mouse model of chronic TB infection, resulting in a 1.9 log_10_ CFU reduction in the lungs after 8 weeks of treatment at 200 mg/kg.^72^ In 2019, Zhang et al. enhanced the metabolic stability of the ester bond in TAM1 through molecular cyclization, resulting in the formation of a tetracyclic, naturally occurring coumestan scaffold. This application of 2° scaffold hopping and further SAR led to the discovery of derivative **7** (Figure 6a), which inhibits PKS-13. The new derivative **7** exhibited approximately 2.3 times longer half-life upon treatment with HLMs than TAM16 and demonstrated 8-fold higher bioavailability *in vivo* in a BALB/c serum inhibition assay at a dose of 100 mg/kg, compared to TAM16 at the same dose. Derivative **7** also displayed efficacy against *Mtb* H_37_Rv (MIC_90_ = 0.011 μM) and a favorable safety profile, as indicated by the selectivity index (SI), which compares the MIC value to cytotoxicity (CC_50_ Vero = 11 μM; SI = 1000). Moreover, **7** proved effective in a mouse model of acute TB infection, yielding a 2.2 log_10_ CFU reduction after 4 weeks of treatment at a dose of 50 mg/kg. However, in a mouse model of chronic TB infection, **7** exhibited only moderate efficacy (0.3 log_10_ CFU reduction after 8 weeks of treatment at dose of 25 mg/kg). In the same experiment, nevertheless, coadministration of **7** with RIF resulted in an additional 0.6 log_10_ CFU reduction compared to RIF alone.^73,75^

The binding pocket of the TE domain of PKS-13 is delineated by the residues Asp1644, Phe1670, Tyr1674, and Asn1640. Based on the cocrystal structure of TAM1 and PKS-13 (PDB ID: 5V3X), it is evident that the benzofuran scaffold of TAM1 is in close proximity to the residue Phe1670, while the hydroxyl group on the benzofuran is positioned at a suitable distance to form a hydrogen bond with Asn1640. The substituted piperidine moiety is located in the vicinity of Tyr1674, facing toward the catalytic site formed by residues His1699 and Ser1533. The phenyl group attached to the 2-position of the benzofuran is near Gln1633 (Figure 6b). According to the crystal structure of TAM16 and PKS-13 (PDB ID: 5V3Y), TAM16 shares the same binding mode as TAM1, with the tertiary amino group in the piperidine ring oriented toward the catalytic site, forming an additional hydrogen bond with Asp1644. The introduction of a hydroxyl group at the 4-position of the phenyl ring attached to the 2-position of the benzofuran enabled the formation of a hydrogen bond with Gln1633 (Figure 6b), which contributed to a significant enhancement of TAM16s activity against *Mtb*.^72–74^ The docking results of **7** (PDB ID: 5V3Y) indicated a highly similar binding mode to that of TAM16, with the top-scored pose displaying the same binding interactions for the coumestan derivative **7** as observed for TAM16. These interactions are characterized by hydrogen bonds with Asp1644, Asn1640, and Gln1633, as well as proximity to Phe1670 and Tyr1674.^73^

Ongoing research identified a cyclic analogue of TAM16, compound **8** (Figure 6a), endowed with a tetracyclic lactam structure. The derivative **8** exhibited activity against *Mtb* H_37_Rv (MIC ranged from 0.085 to 0.17 μM) and demonstrated a satisfactory SI (IC_50_ Vero = 11 μM, SI = 65–129). Thermal shift assay (nanoDSF) suggested that **8** bearing 5*H*-benzofuro­[3,2-*c*]­quinoline-6-one scaffold, also inhibits PKS-13.^76^

A class of 3,5-dinitrobenzamides (DNBs) known to inhibit DprE1 can be considered as open analogues of the structurally related and highly potent BTZs.^58,77^ Initially, Li et al. aimed to simplify the structure of the clinical candidate PBTZ-169 by opening the thiazinone ring within the BTZ scaffold. This modification led to the formation of a 3-nitro-5-(trifluoromethyl)­benzamide scaffold bearing various *N*-alkylated linkers (*N*-oxyethyl **9a**–**e**; *N*-(2-aminoethyl) **10**; *N*-benzyl **11**), and 3,5-dinitrobenzamide scaffold (**12**) was also established (Figure 7). An MIC-based SAR analysis of 37 novel open derivatives of the parent compound PBTZ-169 identified derivative **12**, bearing 3,5-dinitrobenzamide scaffold. This compound displayed strong *in vitro* activity against both DS *Mtb* H_37_Rv (MIC = 0.14 μM) and MDR strains (MIC = 0.035–0.17 μM). Additionally, compound **12** displayed an improved PK profile in mice compared to PBTZ-169 and exhibited a favorable *in vivo* safety profile in a murine acute toxicity model, with all five animals surviving oral administration at a dose of 50 mg/kg, suggesting the promise of this chemical class for further development in TB drug research.^78^

**Figure 7:**
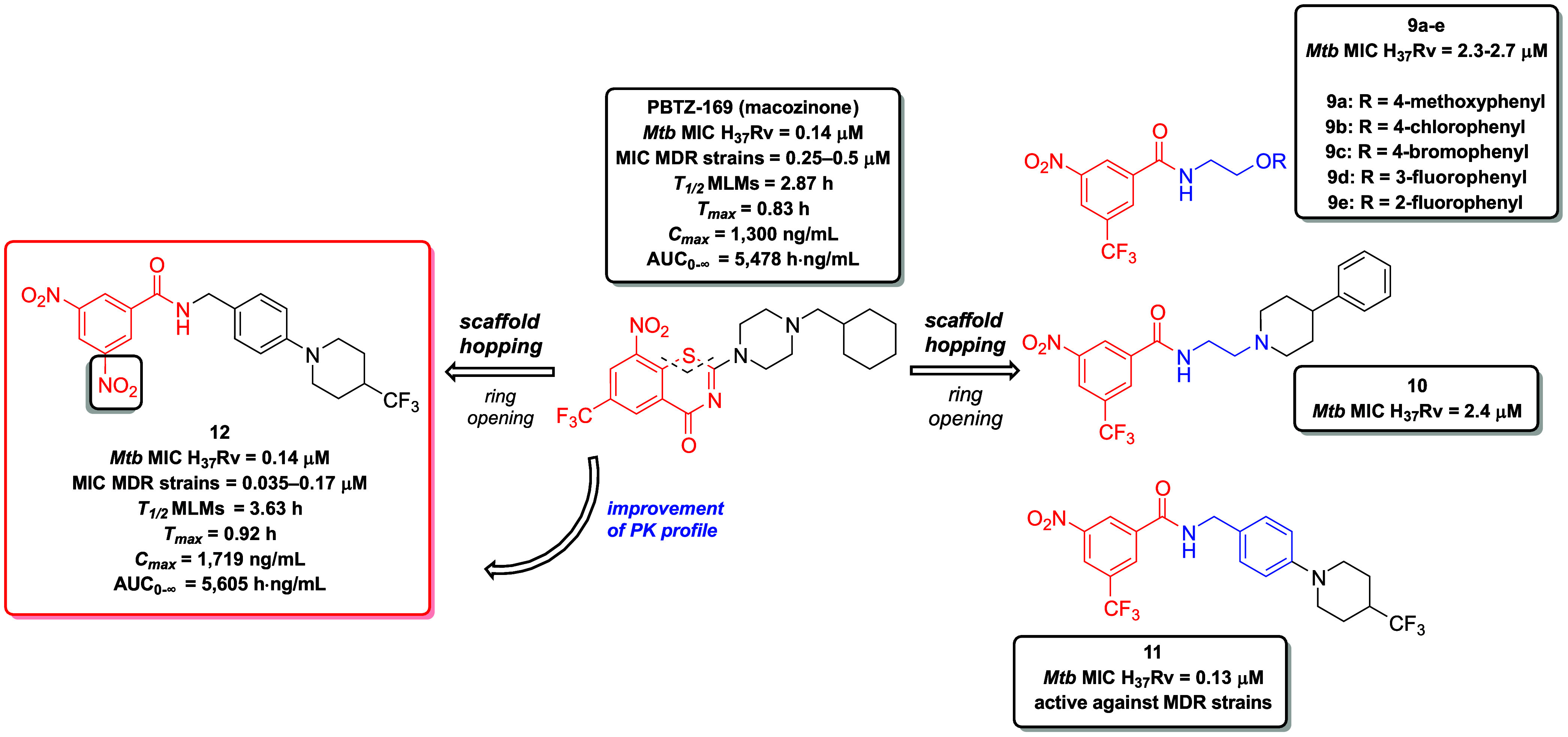
Molecular simplification of the clinical candidate PBTZ-169 leading to the discovery of novel derivatives **9a**–**e**, **10**, **11** and **12** related to the class of DNBs as DprE1 inhibitors. The opening of the thiazinone ring within the benzothiazinone scaffold of PBTZ-169 resulted particularly in the identification of derivative **12**, featuring an N-benzyl linker as an open analog of PBTZ-169. Derivative **12** exhibited an improved PK profile compared to the parent molecule, along with a highly favorable *in vivo* safety profile.

Mycobacterial ATP synthase is a crucial enzyme involved in oxidative phosphorylation, which is responsible for ATP production. ATP synthesis and the proper functioning of this enzyme are driven by the PMF, generated in the ETC through proton translocation across the membrane.^79,80^

Diarylquinolines, including BDQ (Figure 8a) approved by the FDA and European Medicines Agency (EMA) constitute a potent class of inhibitors targeting *Mtb* F_o_F_1_ ATP synthase.^80^ BDQ was first identified in 2005 through a phenotypic screening against *M. smegmatis* and later recommended by WHO for the treatment of MDR-TB.^81^ Despite its strong efficacy compared to existing anti-TB drugs (MIC_99_ = 0.054–0.216 μM), use of BDQ has been significantly limited by serious adverse side effects. These include: (i) prolonged terminal elimination half-life (terminal *T*_1/2_ = 5.5 months) with tissue accumulation due to its high lipophilicity (cLog*P* = 7.25); (ii) toxicity related to human mitochondrial ATP synthase inhibition (IC_50_ = 0.34 μM); and (iii) inhibition of hERG channel, increasing the risk of cardiotoxicity via prolongation of QT interval (hERG IC_50_ = 1.6 μM).^82^ Significantly, resistance to BDQ coupled with both target-based (*atpE*) and efflux-based (*rv*0678) mutations in *Mtb* has emerged relatively quickly following its clinical use.^10^

**Figure 8:**
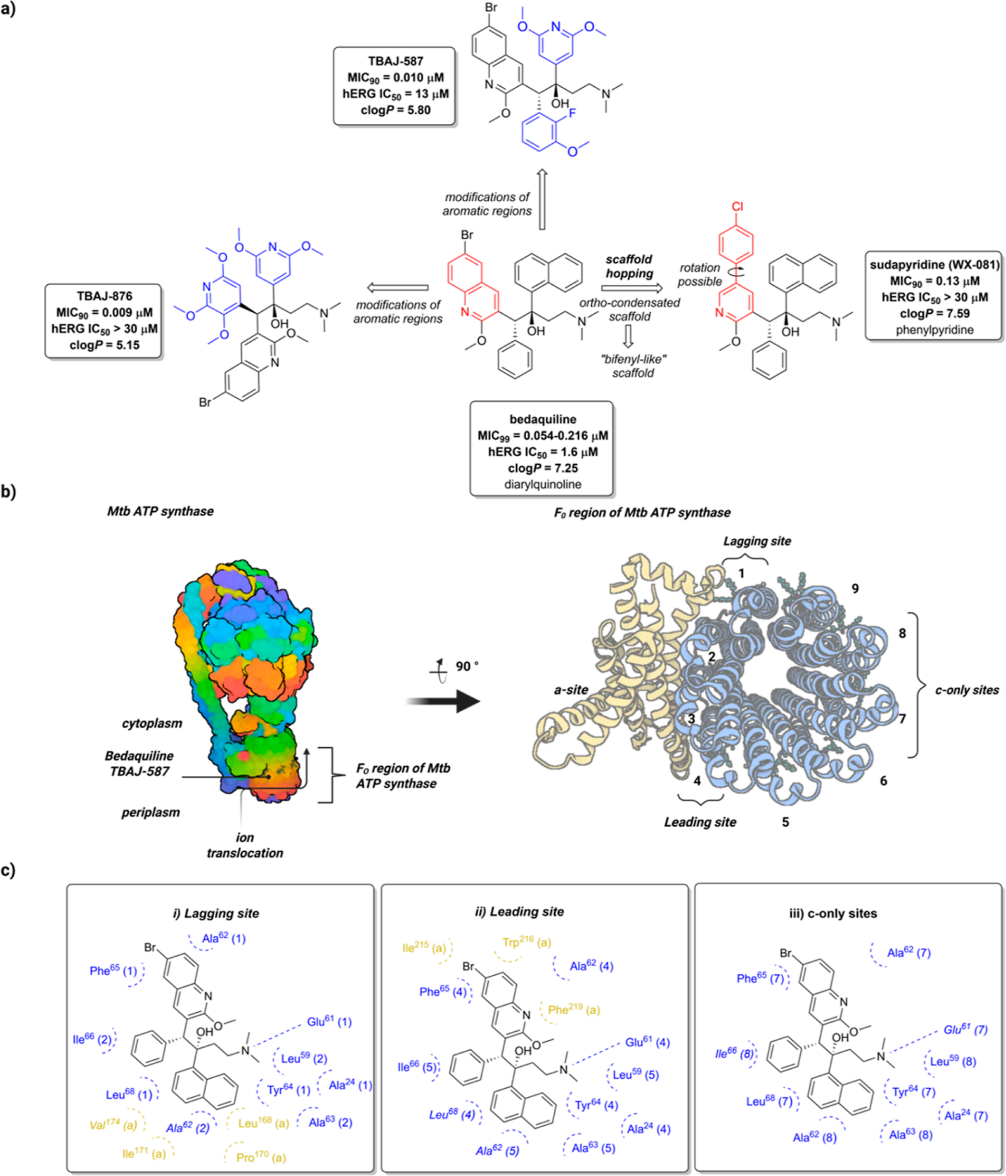
(a) The development pipeline of new inhibitors targeting *Mtb* ATP synthase has been extended from the previously discovered diarylquinoline derivative BDQ, which was approved by FDA and EMA for the treatment of MDR-TB. These modifications of BDQ aimed to mitigate the severe adverse effects associated with BDQ. Structural alterations within the aromatic regions of BDQ led to the discovery of the clinical candidates TBAJ-587 and TBAJ-876. Scaffold hopping, which replaced the quinoline scaffold with less rigid phenylpyridine, resulted in the discovery of sudapyridine (WX-081), currently in phase III clinical trials. (b) ATP synthase is a membrane-protein complex composed of a soluble F_1_ catalytic region and a membrane-embedded F_0_ rotor region, which translocates proton from periplasm to cytoplasm. BDQ occupies the F_0_ rotor region of *Mtb* ATP synthase and restricts its rotation. (c) The binding mode of BDQ involves interactions across three key binding sites within the F_0_ region: (i) the lagging site; (ii) the leading site; and (iii) c-only site, with minor interactions at the a-site residues. TBAJ-587 shared a similar binding mode to BDQ. The H-bond between the c-only sites and BDQ is depicted by a straight, dashed blue line. Other interactions with residues of the c-subunit and BDQ are highlighted in blue, while minor interactions with the a-subunit and BDQ within the lagging site and leading site are illustrated in yellow.

Efforts to reduce lipophilicity and mitigate the inhibitory effect of BDQ to hERG channel through structural modifications in the aromatic regions adjacent to the central quinoline scaffold in BDQ have been the focus of numerous studies. Notably, the successful identification of TBAJ-587 and TBAJ-876 (Figure 8a), both currently undergoing clinical trials, highlights progress in this area.^83,84^ Structural modifications in TBAJ-587 involved the introduction of fluorine at the 2-position and a methoxy group at the 3-position of the phenyl ring, along with the replacement of the naphthalene-1-yl moiety by 2,6-dimethylpyridin-4-yl motif. These alterations not only enhanced its antimycobacterial activity (MIC_90_ H_37_Rv = 0.010 μM) but also reduced the lipophilicity (clog*P* = 5.80) and mitigated cardiotoxicity (hERG IC_50_ > 13 μM). Unfortunately, despite these promising features, TBAJ-587 also exhibits inhibition of human ATP synthase similar to that of BDQ (IC_50_ = 0.50 μM).^80,83^ Further replacement of the 2-fluoro-3-methoxyphenyl group in TBAJ-587 with more hydrophilic 2,3,6-trimethoxypyridin-4-yl resulted in TBAJ-876, with significantly reduced lipophilicity (clog*P* = 5.15) and markedly lower inhibition of hERG channel (hERG IC_50_ > 30 μM), when compared to BDQ, while retaining superior antimycobacterial activity (MIC_90_ H_37_Rv = 0.009 μM).^84^ Generally, the maintenance of excellent activity against *Mtb* in TBAJ-587 and TBAJ-876 is attributed to the preservation of the pharmacophore of BDQ in these diarylquinoline derivatives, specifically the central quinoline scaffold and the adjacent side chains, bearing a tertiary amine group and a tertiary alcohol.^85^

Yao et al. Took an alternative approach to optimize BDQ by modifying the central quinoline scaffold, resulting in the discovery of sudapyridine (WX-081; Figure 8a). This compound is endowed with a 5-(4-chlorophenyl)-2-methoxypyridine moiety with reduced conformational rigidity and is currently undergoing phase III clinical trials.^52,84^ Such structural change can be conceived as atypical ring opening second-degree scaffold hopping. Despite more significant modifications to the original pharmacophore in diarylquinolines, WX-081 exhibited potent activity against *Mtb* (MIC_90_ H_37_Rv = 0.13 μM) and maintained inhibition of mycobacterial ATP synthase. However, its lipophilicity was increased (clog*P* = 7.59) compared to the BDQ and its derivatives. Although WX-081 itself demonstrated reduced cardiotoxicity (hERG IC_50_ > 30 μM), WX-081-related metabolites displayed a similar level of hERG channel inhibition as BDQ metabolite BDQ-M2 (WX-081-M3 hERG IC_50_ = 1.89 μM; BDQ-M2 hERG IC_50_ = 1.73 μM).^85,86^

The mechanism by which BDQ binds to *Mtb* ATP synthase was not fully understood, despite detailed studies on BDQs binding mode with ATP synthase of *M. smegmatis*.^87^ Structural differences between *Mtb* and *M. smegmatis* ATP synthase in their primary sequences indicated that the previously used *M. smegmatis* model was not entirely accurate. This was clarified later on in a study detailing structural insights into the *Mtb* ATP synthase complexed with BDQ and TBAJ-587.^80^ BDQ exhibits a high affinity for *Mtb* ATP synthase, specifically binding to the F_o_ rotor region, which is responsible for an ion shuttling between the periplasm and cytoplasm (Figure 8b). The binding mode of BDQ is characterized by interactions with three distinct binding sites formed by individual subunits within the F_o_ region: the leading site, the c-only site, and the lagging site (Figure 8c). In all three binding sites, the tertiary amino group of BDQ engages with the carboxyl of Glu61 residue from subunit c. Contact with the c-only sites is primarily mediated via hydrophobic interactions with the surrounding residues cAla24, cLeu59, Ala62, cAla63, cTyr64, cPhe65 and cLeu68. At the leading site, where the proton is picked from the periplasm, BDQ interacts with residues Ile215, Trp216, and Phe219 from subunit a and residues from c subunit found at the c-only site. In contrast, the lagging site involves nonpolar residues Leu168, Pro170, Ile171, and Val174 from subunit a and c-only site residues from subunit c. This specific binding mode of BDQ obstructs the rotational function of the F_o_ rotor and inhibits ion translocation between the periplasm and cytoplasm. Although TBAJ-587 exhibited a lower affinity for *Mtb* ATP synthase, its binding mode aligns with the binding mode established for BDQ.^80^

Numerous studies have been published to evaluate the impact of structural modifications on the binding modes within the diarylquinoline series. However, such studies remain elusive for WX-081, a pyridine analog of BDQ discovered by scaffold hopping. Consequently, it is unclear whether the binding mode of WX-081 fully aligns with that of diarylquinolines. Nevertheless, given the moderate structural changes in the central heterocycle and maintaining the key pharmacophoric features in BDQ along with the strong antimycobacterial activity of WX-081, the binding mode of WX-081 most likely remains similar to BDQ and its quinoline analogs.

#### 3° Scaffold Hopping

Third-degree of scaffold hopping, often referred to as peptidomimetics, aims to modify the primary structure of the original peptides to enhance their drug-like properties. Key objectives of this approach include improving the typically unfavorable PK profile of peptides, mitigating their high susceptibility to degradation caused by metabolically labile amide bonds, susceptibility to acidic environment in stomach, and optimizing ADME characteristics. For instance, the large size and high molecular weight of peptides hinder membrane permeability in both host organisms and pathogens, posing a significant challenge in the development of drugs for TB treatment. Another major obstacle for peptide-based drugs is their often-limited selectivity for specific enzymes or receptors arising from their relatively flexible backbones. Despite these limitations, peptide-based therapeutics offer various advantages, notably their high efficacy and broad spectrum of biological activity.^88–92^

The prokaryotic ubiquitin-like protein, *Mtb* 20S, is a multimeric peptidase and core component of the *Mtb* proteasome, found in mycobacteria.^93,94^ *Mtb* 20S degrades damaged and potentially toxic proteins in *Mtb*, which can result from exposure to reactive nitrogen species (RNS). This system serves as an alternative to the ubiquitin system found in eukaryotes.^95^ Although *Mtb* 20S is not essential for mycobacterial survival *in vitro*, it is crucial for bacterial survival within the mammalian host, where *Mtb* is exposed to RNS produced by innate immune cells, such as macrophages.^96,97^ Targeting this enzyme offers a novel therapeutic strategy that focuses not on directly killing *Mtb* but on sensitizing it to the host immune system. Given the structural and catalytic similarities between the essential human 20S proteasome (Hu 20S) and *Mtb* 20S, drugs designed for this strategy must exhibit high selectivity for *Mtb* 20S.^98^

Selectivity for drugs primarily active against *Mtb* 20S can be achieved by exploiting structural differences and binding site preferences unique to this 20S peptidase. Both eukaryotic and prokaryotic proteasomes share a barrel-shaped structure composed of two outer rings formed by α subunits and two inner rings of catalytically active β subunits, where proteolysis takes place at the N-terminal threonine residues. In eukaryotes, there are seven types of α subunits and seven types of β subunits, with only β_1_, β_2_, and β_5_ being catalytically active. In contrast, *Mtb* possesses catalytically active β subunits of a single type. The S1 and S3 pockets of the β subunits in *Mtb* 20S display a preference for bulky tryptophan residues at the P1 position in peptide-based drugs and glycine or proline residues at the P3 position.^94^ In light of these structural preferences, several peptides have been identified that selectively inhibit *Mtb* 20S. These peptides were derived from anticancer drugs that inhibit the Hu 20S, achieving selective inhibition of *Mtb* 20S through modifications of the primary structure at either the P1 or the P1 and P3 positions.^94,98^

To enhance selectivity for *Mtb* 20S over Hu 20S, Lin et al. studied structural modifications at the P1 position of the tripeptide-based anticancer drug bortezomib (Figure 9a), which displays strong affinity and inhibitory potency toward the β_5_ subunit of the Hu 20S. Among the 18 P1 amino acids analogs of bortezomib, the most potent structure was derivative **13** (Figure 9a), bearing a 3-chlorophenyl moiety at the P1 position to mimic the preferred bulky tryptophan. The compound **13** demonstrated a submicromolar activity against both the chymotryptic-like (*Mtb20SOG* (Ac-RFW-AMC) IC_50_ = 0.15 μM) and tryptic-like (*Mtb20SOG* (*Z*-VLR-AMC) IC_50_ = 0.13 μM) active sites of the β subunits in *Mtb* 20S, and a 75-fold reduction in affinity for the β_5_ subunit of Hu 20S compared to bortezomib (Hu 20S IC_50_ = 1.2 μM for compound **13** vs 0.016 μM for bortezomib).^94^

**Figure 9:**
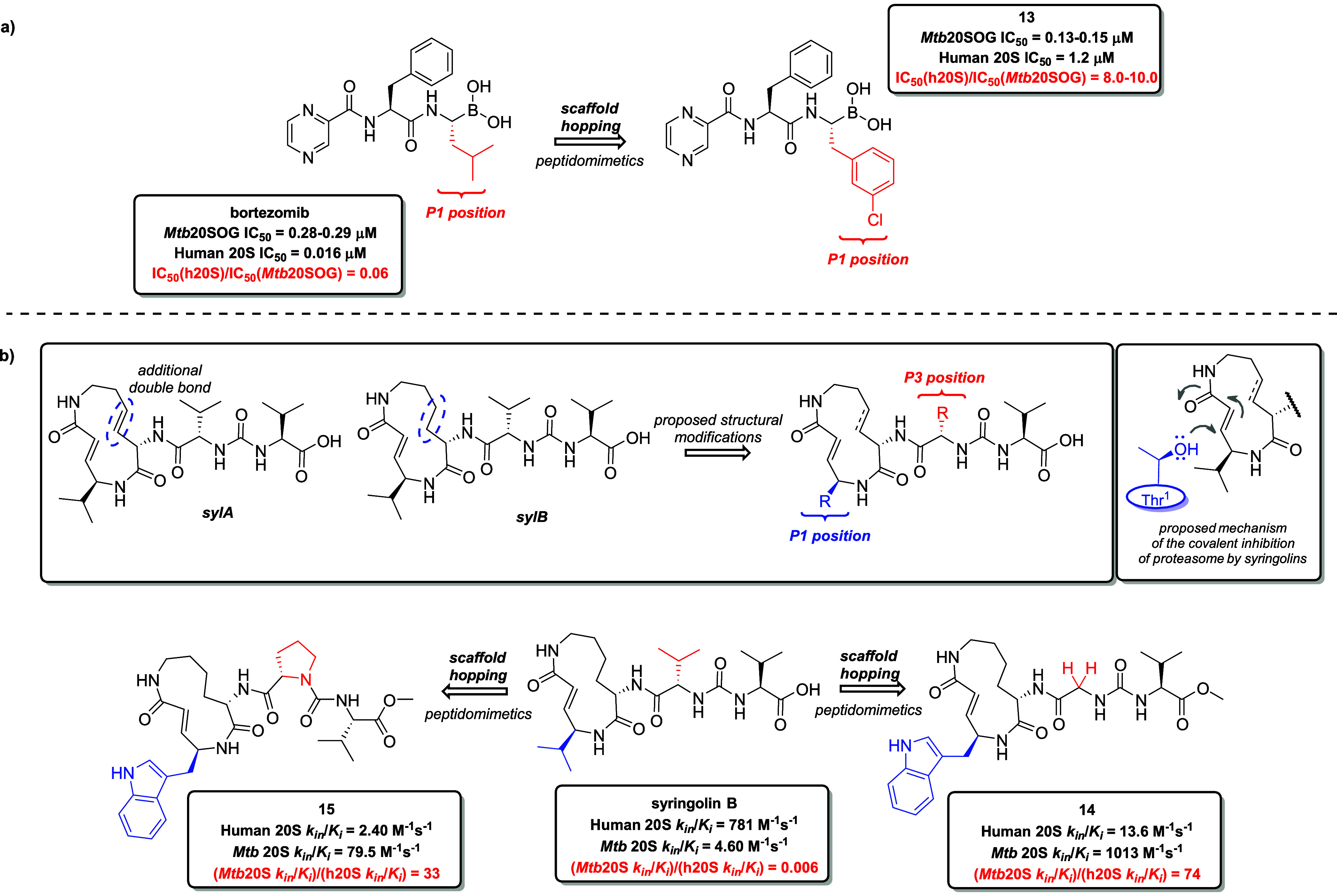
Application of 3° scaffold hopping in the development of TB therapeutics: (a) modification of the primary structure at the P1 position of the tripeptidyl boronate drug bortezomib, a human 20S inhibitor, to align with the structural preferences of the S1 binding pocket of the β-subunit of the *Mtb* 20S proteasome. This approach led to the discovery of derivative **13**, a structure that mimics the original bortezomib while achieving high selectivity for *Mtb* 20S proteasome. (b) Modification of the primary structure of naturally occurring syringolins (sylA and sylB) at both P1 and P3 positions designed to match the structural preferences of the S1 and S3 pockets of the β-subunits of the *Mtb* 20S. This effort resulted in the development of derivatives **14** and **15**, preserving the original activity of syringolins, while exhibiting high selectivity for the *Mtb* proteasome.

Building on the previous findings, Totaro et al. pursued the development of novel peptide-based compounds that selectively inhibit *Mtb* 20S. To establish a suitable parent molecule as a starting point, the researchers investigated peptidomimetic 20S inhibitors with documented anticancer activity, particularly emphasizing those molecules exerting a covalent inhibition mechanism. This mechanism was selected to prevent potential off-target interactions, as seen in bortezomib.^98,99^ Syringolins, especially syringolin A and B, are natural products that meet these criteria, having an acrylamide motif integrated into their terminal macrolactam structure. Such structural moiety allows irreversible binding to the threonine residue of the proteasome β subunit, critical for proteolytic activity, through a Michael-type addition.^98^ Here we note that the author‘s preference for covalent inhibition of the mentioned syringolin class, potential *Mtb* 20S inhibitors is rather unusual. For instance, the acrylamide motif functions as nonspecific Michael acceptor and, according to Brenḱs criteria, represents a structural alert. Covalent inhibitors containing a Michael acceptor within their structure can thus interact with nucleophilic biomolecules, particularly proteins bearing cysteine residues, potentially leading to undesirable off-target interactions.^100,101^ Unlike the aforementioned study, which inspected modifications of bortezomib at the P1 position only,^94^ it was further hypothesized that single-point modifications at both the P1 and P3 positions within the syringolins backbone could lead to even enhanced selectivity for *Mtb* 20S.^98^

The efficacy of the newly discovered syringolins was evaluated through kinetic enzymatic assay. Potency was quantified as the ratio of second-order rate constants for inhibition, *k*_in_/*K*_i_, determined from the enzyme-catalyzed hydrolysis of a fluorogenic substrate. The initial series of syringolin-mimicking peptides focused on structural modifications at the P1 position of syringolin B. Based on earlier insights into the S1 pocket’s preference for bulky substituents within the *Mtb* 20S β subunit, aromatic substituents were selected to replace the original isopropyl group in syringolin B at the P1 position. Surprisingly, the introduction of various substituted phenyl residues led to increased affinity of these P1 amino acids analogs of syringolin B toward Hu 20S, contrary to prior findings and trends observed in structural modifications of bortezomib. Incorporating a indol-3-yl methyl motif as a tryptophan side chain significantly enhanced activity against *Mtb* 20S, corresponding well with earlier results from the study by Lin et al.^94^ The methylindole moiety was thus fixed at the position P1 for further structural modifications, directing toward modifications at the P3 position. Indeed, certain substitutions at P3 proved essential for enhancing selectivity toward *Mtb* 20S, with derivatives bearing either a glycine (derivative **14**) or proline residue (derivative **15**) at P3 demonstrating the highest selectivity for *Mtb* 20S (Figure 9b). In conclusion, the authors synthesized an altered syringolin A that differs from template syringolin B by the additional double bond within the macrolactam ring. This modified structure incorporates the most effective substituents of derivative **14** at positions P1 and P3. However, this peptide was highly reactive and nonselective toward *Mtb* 20S. Given the common challenges associated with the unfavorable ADME properties of peptide-based drugs,^90^ the two most potent derivatives **14** and **15** were tested for their ability to penetrate the cell wall and sustain the whole-cell activity using *Mycobacterium bovis* as a model organism for *Mtb*. Accordingly, the proteasome activity was assessed following whole-cell lysis, defined as the percentage of the original activity in untreated *M. bovis* (control group with 100% activity). Both derivatives **14** and **15** demonstrated a significant degree of whole-cell activity, coupled with their capability to penetrate the cell wall of *M. bovis*, showing inhibition in the range of approximately 40–70% (at 20 μM). While derivative **14** showed greater potency in early assays, derivative **15** demonstrated stronger inhibitory activity in whole-cell experiments. The efficacy of derivative **15** was attributed to the higher lipophilicity given the presence of proline residue at the P3 site compared to derivative **14** bearing glycine residue.^98^ Nevertheless, it is crucial to note that the results from biological assays conducted using *M. bovis* as a surrogate for *Mtb* cannot be automatically extrapolated to *Mtb*.^102^

DPLG-2 (Figure 10a) is a noncovalent dipeptide proteasome inhibitor belonging to the N,C-capped dipeptide family, identified via HTS and subsequently optimized through structural single-point alterations at the P1, P2, P3, and P4 positions within the primary peptide backbone.^103^ DPLG-2 exhibits high potency and remarkable selectivity for the *Mtb* 20S over human constitutive proteasome (Hu c-20S) and immunoproteasome (Hu i-20S). Importantly, DPLG-2 displayed bactericidal activity against nonreplicating *Mtb* under nitrosative stress conditions.^103^ The selectivity of this dipeptide arises primarily from structural differences between the *Mtb* 20S and Hu 20S in the S1 and S3 substrate-binding pockets, which have been confirmed by X-ray analysis using the molecular replacement method (PDB ID: 3HFA).^103,104^ In particular, the bulky and rigid P1 substituent, a naphthyl group mimicking the preferred tryptophan,^94^ and the presence of a tertiary nitrogen in the P3 position, as a part of the Asn amide side chain, contribute significantly to its selective binding. The tertiary nitrogen is especially important for favorable interactions with the *Mtb* 20S S3 pocket, whereas the Hu 20S exhibit preference for a secondary nitrogen in the P3 position.^103^ The biological activity of DPLG-2 was evaluated using multiple experimental approaches: IC_50_ determination using fluorogenic chymotryptic substrate (Suc-LLVY-AMC) revealed a value of 0.015 μM for *Mtb*20SOG and SI index over 4700 and 3600 for Hu c-20S and Hu i-20S, respectively. In bactericidal assays, DPLG-2 reduced CFUs of *Mtb* H_37_Rv in a dose-dependent manner under nitrosative stress conditions.^103^ Furthermore, DPLG-2 was capable of penetrating intact *M. bovis* cells.^103,104^ Nevertheless, as noted above, it is important to recognize that results obtained using *M. bovis* as a surrogate model may differ substantially from those observed in studies directly involving *Mtb*.^102^

**Figure 10:**
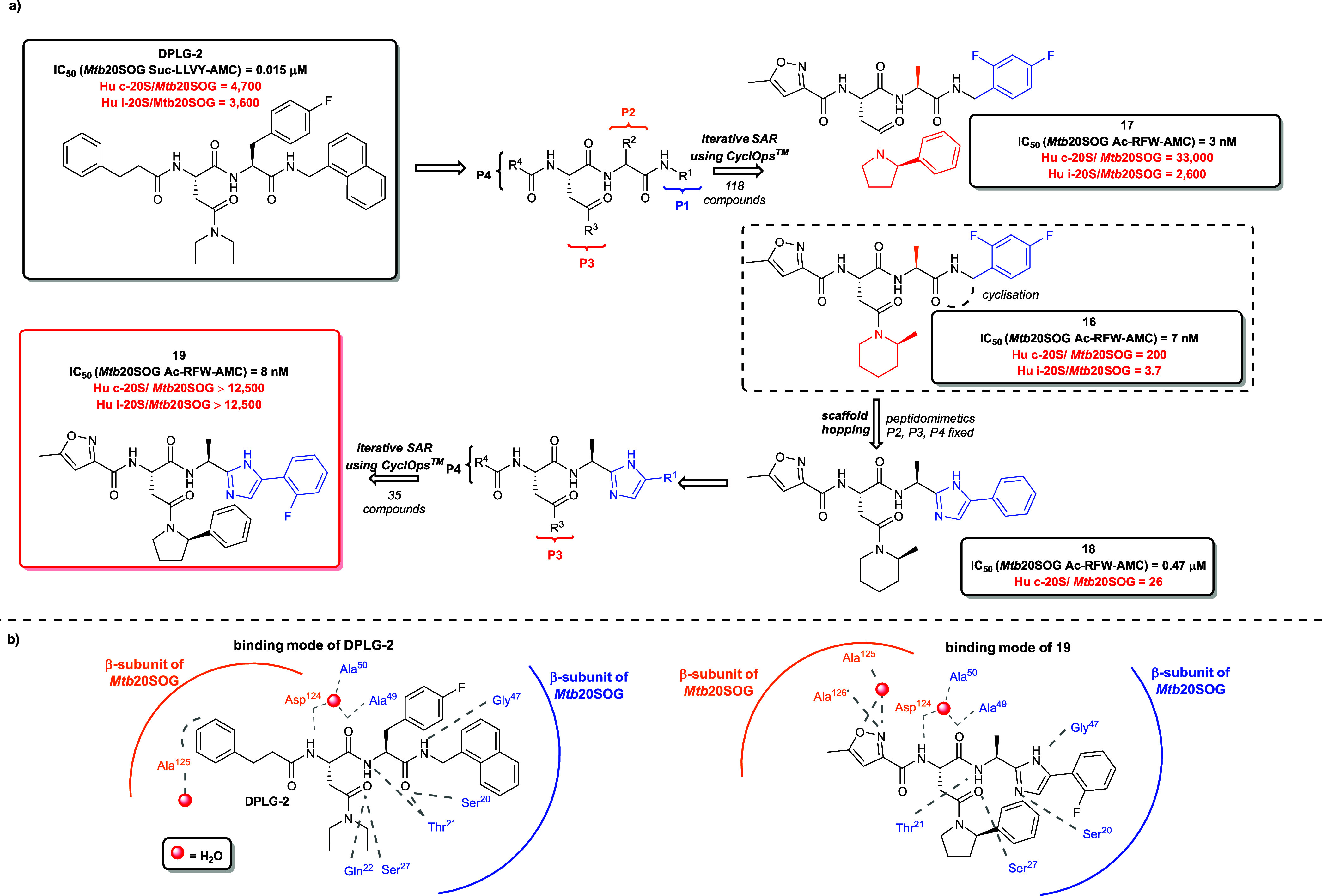
(a) Development pipeline of selective *Mtb* 20S inhibitors derived from the N,C-capped dipeptide DPLG-2, aimed at improving target selectivity and metabolic stability. Iterative single-point modifications at the P1–P4 positions were performed using CyclOps, followed by 3° scaffold hopping at P1 to enhance amide bond stability near the catalytically essential Thr1 residue. Derivative **19** showed improved potency and selectivity against *Mtb* 20S but lacked metabolic stability data and failed to exhibit whole-cell activity against *Mtb* H37Rv, likely due to poor permeability and intracellular biotransformation. (b) X-ray structures of DPLG-2 and **19** (PDB IDs: 5TRG and 6ODE, respectively) revealed conserved binding conformations. In **19**, the amide bond at P1 was replaced by a substituted imidazole, maintaining key H-bonds with Ser20 and Gly47 in the S1 pocket (gray dashed lines). Additional peptide backbone interactions are also shown (gray dashed lines); water molecules are depicted as red spheres.

The binding mode of DPLG-2 (Figure 10b) was determined through X-ray analysis (PDB ID: 5TRG) and primarily involves hydrogen bonding interactions within two adjacent β-subunits of *Mtb* 20S. The substrate specificity is largely attributed to the shape of the S1 and S3 binding pockets of *Mtb* 20S. Within one β-subunit, at the P1 position, the interaction between DPLG-2 and *Mtb* 20S is mediated by hydrogen bonds with residues Gly47, Ser20, and Thr21. The P2 substituent does not form any significant interactions with the S2 pocket of *Mtb* 20S. At the P3 position, the interaction is facilitated by H-bonds with Ser27 and Gln22, with the H-bond to Gln22 being particularly specific to *Mtb* 20S. The binding mode of DPLG-2 is further stabilized by interactions with Ala49 and a water-mediated bridge to Ala50. The second adjacent β-subunit forms a hydrogen bond with Asp124 and a hydrophobic interaction between the phenyl group at P4 and Ala125.^104^

Zhan et al. applied a 3° scaffold-hopping strategy to the dipeptide DPLG-2 (Figure 10a) as part of a structure-based optimization effort aimed at developing novel and selective inhibitors of the *Mtb* 20S with enhanced metabolic stability compared to the parent DPLG-2.^105^ Their approach began with the generation of an iterative SAR through systematic optimization of the P1, P2, P3, and P4 positions within the parent DPLG-2. In total, 118 N,C-capped dipeptides were synthesized using the CyclOps microfluidic platform and screened in parallel against *Mtb* 20S, Hu c-20S, and Hu i-20S. The initial biological evaluation identified N,C-capped dipeptides **16** (Figure 10a; IC_50_ (*Mtb*20SOG Ac-RFW-AMC) = 7 nM; SI = 3.7 and 200 for Hu i-20S and Hu c-20S, respectively) and **17** (Figure 10a; IC_50_ (*Mtb*20SOG Ac-RFW-AMC) = 3 nM; SI ≥ 2600 and >33,000 for Hu i-20S and Hu c-20S, respectively) as a template structures for further development.^105^

The authors performed X-ray crystallography of the *Mtb* 20S active site in complex with inhibitors **16** (PDB ID: 6OCW) and **17** (PDB ID: 6OCZ) to investigate the structural implications of substitutions at the P1 and P3 positions. Both compounds exhibited a binding mode similar to that of DPLG-2. However, a paradoxical observation emerged regarding the P3 substitution: the more selective inhibitor **17**, which contain a bulky 2-phenylpyrrolidin-1-yl moiety at P3, was unable to form the hydrogen bond between the Asn amidic carbonyl and the Gln22 residue; an interaction considered critical and specific for *Mtb* 20S selectivity. In contrast, this key hydrogen bond was preserved in the nonselective inhibitor **16**, which features a 2-methylpiperidin-1-yl group at the same position. Despite this apparent contradiction, the authors selected **16** as the structural template for subsequent 3° scaffold hopping efforts.^105^

3° scaffold hopping was implemented at an advanced stage of this study to enhance the metabolic stability of the parent molecule, reportedly labile dipeptide DPLG-2, via bioisosteric replacement of the amide bond at the P1 position in compound **16** with a 5-phenyl-substituted imidazole, yielding compound **18** (Figure 10a). Although no explicit rationale was provided for targeting this particular amide bond, and despite previous data indicating that DPLG-2 displays high stability in human plasma,^103^ we hypothesize that the P1 amide bond may be particularly susceptible to hydrolysis due to its proximity to the Thr1 residue in S1 active site, which is critical for the proteolytic activity of the *Mtb* 20S.^98^ Inhibitor **18**, a novel peptidomimetic derivative originated from **16**, demonstrated good inhibitory activity against *Mtb* 20S but also moderate activity toward Hu c-20S (IC_50_ *Mtb*20SOG Ac-RFW-AMC = 0.47 μM); SI (Hu c-20S = 26).^105^ Compound **18** was subsequently selected as a template for further structural optimization, focusing on modifications of the phenyl ring attached to the imidazole moiety at the 5-position in the P1 region of the peptide, as well as variations at P3 and P4. These optimizations were guided by an iterative SAR approach using the CyclOps and screening platform. Among 35 newly synthesized N,C-capped peptides bearing the 5-phenylimidazole motif, compound **19** (Figure 10a) emerged as a potent *Mtb* 20S inhibitor with excellent selectivity (IC_50_ *Mtb*20SOG Ac-RFW-AMC = 8 nM; SI > 12,500 for both Hu i-20S and Hu c-20S), surpassing that of the parent compound DPLG-2 (IC_50_ (*Mtb*20SOG Suc-LLVY-AMC) = 0.015 μM; SI = 3600 and 4700 for Hu i-20S and Hu c-20S, respectively).^103–105^ However, it is important to critically note that although the authors applied a logical and well-reasoned strategy resulting in the discovery of highly selective *Mtb* 20S inhibitors, the study lacks experimental validation of the metabolic stability of the newly synthesized peptidomimetics and their comparison with the parent compound DPLG-2, which—according to the initial claims—was the primary objective of this comprehensive investigation. Additionally, the authors report that, in subsequent phases of biological evaluation, the newly developed 5-phenylimidazole derivatives of DPLG-2 demonstrated no significant activity against *Mtb* H_37_Rv in whole-cell screening assays. This lack of efficacy was presumably attributed to unfavorable ADME properties, particularly poor cell membrane permeability or undesired intracellular biotransformation.^106^

The binding mode of the novel phenylimidazole derivative **19** (Figure 10b) was validated by X-ray crystallography (PDB ID: 6ODE). Bioisosteric replacement of the amide bond present in both DPLG-2 and its analogue **16** bearing a 5-phenylimidazole moiety resulted in retention of the overall binding conformation of compound **19**, consistent with that observed for DPLG-2 and derivative **16**.^104,105^ The 5-phenylimidazole moiety in **19** maintains key interactions within the S1 binding pocket of *Mtb* 20S, including hydrogen bonds with Gly47 and Ser20, analogous to those established by DPLG-2. Notably, this bulky aromatic system in **19** cannot be accommodated within the S1 pocket of Hu c-20S or Hu i-20S, which likely accounts for the exceptional selectivity of **19** toward *Mtb* 20S. The binding interactions within the S2 and S4 pockets remain unaltered compared to DPLG-2 and compound **16**. Interestingly, molecule **19** also carries the sterically demanding 2-phenylpyrrolidin-1-yl substituent at the P3 position, which prevents the formation of a hydrogen bond with the *Mtb*-specific Gln22 residue. This observation suggests that the interaction with Gln22 may be less critical for selective *Mtb* 20S inhibition than previously assumed, and that other interactions—particularly those involving the S1 pocket—play a more dominant role in driving selectivity.^105^

Macrocyclization is one of the strategies encompassed by peptidomimetics. This approach modulates the conformational flexibility of peptide backbones, whereby the formation of a macrocycle from an originally flexible peptide chain results in a more rigid structure, directly influencing the potency, selectivity and physicochemical properties of peptide-based therapeutics.^92^

Building upon previous work, Zhang et al. explored an alternative peptidomimetic strategy applied to the N,C*-*capped dipeptide DPLG-2 ^103^ (Figure 11a) and its phenylimidazole-based derivatives,^105^ which exhibited suboptimal whole-cell activity against *Mtb* H_37_Rv. As a divergent approach, the authors implemented a macrocyclization strategy, covalently linking the P4 and P2 positions of DPLG-2 led to the formation of macrocyclic analogs with varying ring sizes and reduced conformational flexibility (Figure 11a). The rationale for such modification was based on conformational analysis, which revealed that the P2 and P4 substituents are in close spatial proximity even in the acyclic conformation of DPLG-2 (Figure 11a).^103,104,106^ This hypothesis was further supported by structural data showing that the most critical binding interactions with the *Mtb* 20S are mediated through the P1 and P3 positions.^106^ Thus, the P2 and P4 regions were deemed suitable targets for structural optimization aimed at enhancing the potency and potentially the drug-like properties of these peptidomimetic inhibitors.^106^ Macrocyclization was hypothesized to enhance the pharmacological properties of DPLG-2-based peptide inhibitors and was initially assessed using molecular docking. The cocrystal structure of the *Mtb*20SOG (PDB ID: 5TS0) was employed in the study, wherein the 4-fluorobenzyl group at the P2 position of DPLG-2 was removed, and the P2 and α-position of P4 in DPLG-2 were covalently tethered via aliphatic or ether-based linkers, accompanied by subsequent structural simplification at the α-position within the P4 (further highlighted as P5; Figure 11a).^106^ This strategy yielded macrocyclic peptides containing 14- to 17-membered rings. Comparative binding mode analysis revealed that 15- and 16-membered macrocycles adopted conformations closely resembling that of the parent compound DPLG-2. Among these, macrocycle **20** (Figure 11a) was selected for further development due to its most favorable inhibitory activity against *Mtb* 20S (IC_50_ (*Mtb*20SOG Suc-LLVY-AMC = 0.10 μM)) combined with the highest selectivity over Hu c-20S and Hu i-20S (SI > 980 and >980, respectively).^106^

**Figure 11:**
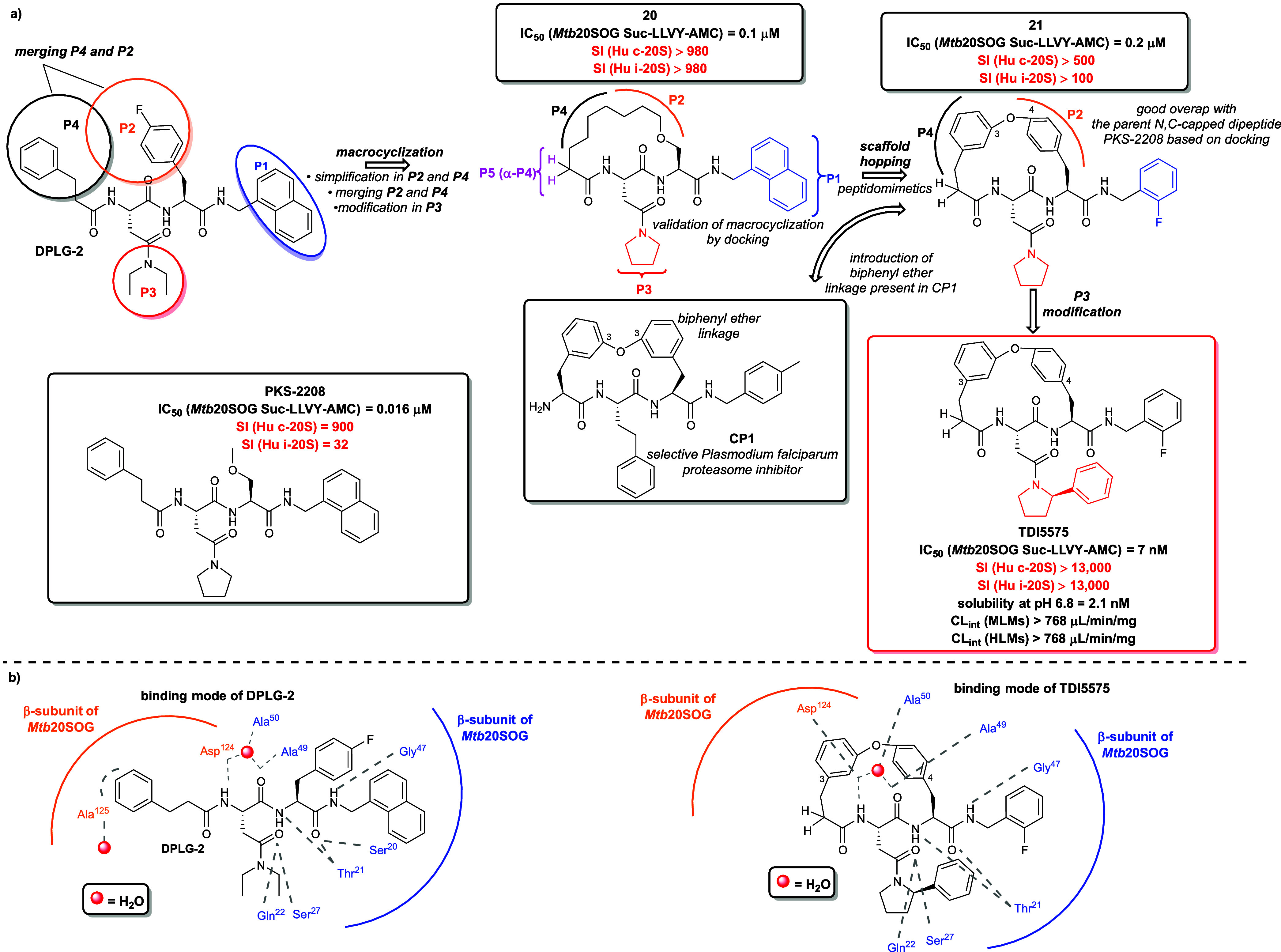
(a) A macrocyclization strategy was applied in the development of potent and selective inhibitors of *Mtb* 20S, derived from the parent N,C-capped dipeptide DPLG-2. In the initial stage of this optimization process, the macrocyclization hypothesis was validated using in silico molecular docking studies on a series of 14- to 17-membered macrocyclic derivatives of DPLG-2, leading to the identification of a model macrocycle **20**. In the subsequent step, a 3,4-biphenyl ether bridge—previously reported in the *Plasmodium falciparum* inhibitor CP1—was introduced between the P2 and P4 positions of compound **20**. This modification yielded the novel macrocycle **21**, which was further structurally refined to enhance selectivity toward *Mtb* 20S, ultimately leading to the macrocyclic peptidomimetic TDI5575. TDI5575 demonstrated excellent inhibitory potency, high selectivity toward *Mtb* 20S over the Hu 20S, and significant whole-cell antimycobacterial activity under nitrosative stress. However, metabolic stability studies using HLMs and MLMs revealed notable liabilities in terms of biotransformation susceptibility, accompanied by suboptimal aqueous solubility. (b) The binding mode of TDI5575 (PDB ID: 6WNK) was elucidated via X-ray analysis, revealing a high degree of spatial alignment with the parent dipeptide DPLG-2 (PDB ID: 5TRG). TDI5575 interacts with *Mtb* 20S at the interface of two adjacent β-subunits, predominantly through H-bonds, depicted as gray dashed lines, with the S1 and S3 binding pockets. Key interacting residues include Gly47, Thr21, Gln22, Ser27, and Ala50, with an additional water-bridged interaction involving Ala50 within one β-subunit. The binding pose is further stabilized by a H-bond with Asp124 in the neighboring β-subunit.

Based on the validated hypothesis that macrocyclization can enhance the potency of N,C-capped dipeptides, the same research group adopted 3° scaffold hopping aimed at structurally modifying the ether linker connecting the P2 and P4 positions within macrocycle **20** (Figure 11a). Specifically, the ether linker in compound **20** was replaced with a biphenyl ether tether, and the naphthyl moiety at the P1 in macrocycle **20** was replaced with a 2-fluorobenzyl group.^106^ Notably, the biphenyl ether linker connecting the P2 and P4 positions is also present in the previously reported peptide CP1 (Figure 11a), which was developed as a selective inhibitor of the *Plasmodium falciparum* proteasome.^107^ Despite CP1 showing no inhibitory activity against *Mtb* 20S, likely due to a nonoptimal substitution at the P3 position (homophenylalanine),^106^ CP1 shares a highly similar peptide backbone with N,C-capped dipeptides known to selectively inhibit *Mtb* 20S.^103,105^ A series of macrocyclic peptides originated from macrocycle **20** and CP1 was subjected to docking studies using the cocrystal structure of *Mtb*20SOG (PDB ID: 5TS0). Among the analogues, macrocycle **21** (Figure 11a; IC_50_ (*Mtb*20SOG Suc-LLVY-AMC) = 0.20 μM; SI > 100 and >500 for Hu i-20S and Hu c-20S, respectively), incorporating a 3,4́-biphenyl ether tether and a pyrrolidin-1-yl group at the P3 position, exhibited the highest docking score and a binding mode highly similar to that of the parent N,C-capped dipeptide PKS-2208 (Figure 11a).^104,106^ This binding mode revealed key interactions with two β-subunits of the *Mtb* 20S, involving hydrogen bonds with residues Thr21, Gly47, Ala49, Asp124, Gln22, and Ser27.^106^

Final derivatization of the macrocycle **21** at the P3 position, guided by docking, involved the introduction of a phenyl at position 2 within the pyrrolidine present in **21**. This structural modification led to the identification of a macrocycle designated TDI5575 (Figure 11a), which demonstrated excellent inhibitory activity and high selectivity toward the *Mtb* 20S (IC_50_ (*Mtb*20SOG Suc-LLVY-AMC = 7 nM, SI > 13,000 for both Hu c-20S and Hu i-20S)).^106^ TDI5575 was also able to penetrate *Mtb* and inhibit protein degradation mediated by the *Mtb* 20S, as indicated by the accumulation of pupylated proteins, leading to bacterial cell death under nitrosative stress conditions.^106^ TDI5575 exhibited a favorable safety profile, as indicated by 98% viability of HepG2 cells following 72 h incubation with TDI5575 at 30 μM. Furthermore, TDI5575 displayed low solubility (2.1 nM) at pH 6.8—the pH value simulating the environment of inflamed tissue or an infected macrophage. However, this solubility value thus lies below the effective concentration of TDI5575 (IC_50_ = 7 nM) against *Mtb* 20S. Subsequent studies focusing on metabolic stability revealed that TDI5575 was stable in human plasma, with 94% of the compound remaining after 2 h. However, TDI5575 was rapidly metabolized (CL_int_ > 760 μL/min/mg in both HLMs and MLMs). Additionally, TDI5575 demonstrated high passive permeability as assessed by the parallel artificial membrane assay (PAMPA permeability = 227 nm/s).^106^Herein we should note that PAMPA assay is not entirely suitable for evaluating peptide drug permeability across biological membranes, as it neglects active transport mechanisms. Many peptides are absorbed via carrier-mediated or receptor-mediated processes, which PAMPA does not consider. More appropriate methods represent Caco-2 cell assays or in vivo models that reflect enzymatic and transporter interactions.^108^

The binding mode of TDI5575 (Figure 11b; PDB ID: 6WNK) was validated by X-ray analysis using the molecular replacement method, employing the DPLG-2-bound *Mtb* 20S structure (PDB ID: 5TRG) as a search model.^106^ The binding mode of TDI5575 closely resembled that of DPLG-2. TDI5575 adopts a similar conformation to the acyclic DPLG-2 within the *Mtb* 20S. Interactions of TDI5575 involve residues from both adjacent β-subunits of *Mtb* 20S. Within one β-subunit, key interactions occur at the S1 and S3 pockets, where the P1 and P3 moieties of the TDI5575 form hydrogen bonds with residues Thr21, Gly47, and Ala49. Despite the steric hindrance of the 2-phenylpyrrolidin-1-yl at the P3 position in TDI5575, the carbonyl group at P3 of TDI5575 can engage in hydrogen bonding with Gln22 and Ser27. This observation suggests that macrocyclization induces a conformational adaptation that enables this interaction, in contrast to the acyclic dipeptide **19** (Figure 10a), which bears the same bulky P3 moiety, sterically prevents interaction with the Gln22 side chain and the P3 carbonyl of **19**.^105^ Additionally, the binding mode of TDI5575 is stabilized by interaction with residue from the second β-subunit, specifically Asp124, including a water-mediated hydrogen bond bridge between Ala50 and Asp124.^106^

#### 4° Scaffold Hopping

4° Scaffold hopping involves the most extensive and significant alterations to the core scaffold, generating a completely new chemical entity. Given that more pronounced structural modifications to the parent molecule inherently carry a higher risk of failure, this type of scaffold hopping is relatively underreported in the literature, and the development of TB therapeutics is no exception. While this type of scaffold hopping often overlaps with VS, it is crucial to emphasize that in this context, VS serves as a tool to facilitate this advanced level of scaffold hopping. In contrast to conventional VS, which identifies entire molecules as hits, this approach focuses solely on identifying an alternative core that is compatible with the existing molecular structure.^33^

Like TBA-7371 and quabodepistat, the TCA1 (Figure 12a) contains a thiophene carboxamide moiety, representing another noncovalent DprE1 inhibitor. TCA1 was identified through cell-based phenotypic screening and exhibited bactericidal effects against both replicating and nonreplicating *Mtb*.^109^ Wang et al. aimed to discover new derivatives of TCA1 to enhance its biological activity and drug-like properties. The study began with an analysis of the cocrystal structure of DprE1 in complex with TCA1 (PDB ID: 4KW5), revealing that the 2,3-disubstituted thiophene, which constitutes the pharmacophore of TCA1, primarily engages in hydrophobic interactions with residues Lys367 and Asn385 within the active site of DprE1. The thiophene sulfur atom also interacts with His132, further stabilizing the binding within DprE1’s active site. Furthermore, the carboxamide linkage between the thiophene and benzothiazole forms a hydrogen bond with Lys418. The benzothiazole is oriented parallel to the isoalloxazine ring in FAD, forming additional hydrophobic interactions with residues Cys387, Tyr314, Gln334, and Tyr60. The binding mode of TCA1 is further supported by additional interactions from the imido ester side chain at position 3 of the thiophene, which includes a hydrogen bond between one imido ester carbonyl and Ser228, along with hydrophobic interactions involving Trp230, Lys134, and Val365 (Figure 12b). These observations led the researchers to hypothesize that introducing an additional hydrogen bond acceptor in benzothiazolés region could further enhance DprE1 binding affinity.^109,110^

**Figure 12:**
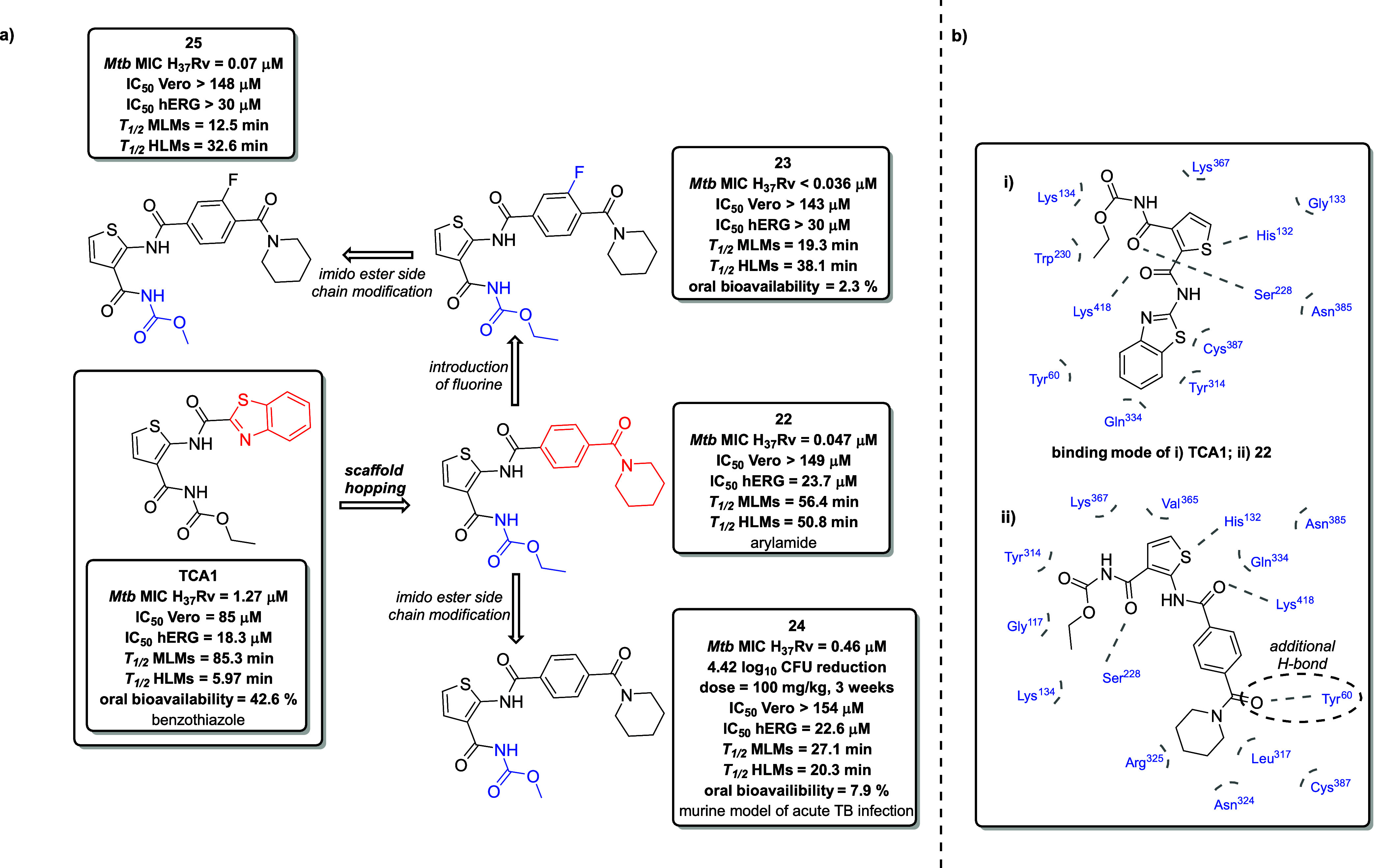
(a) 4° Scaffold hopping applied to TCA1 to enhance affinity for the DprE1 active site. Scaffold hopping was applied to TCA1 to improve its binding affinity to the DprE1 active site by introducing a H-bond acceptor within the benzothiazole region of TCA1. This effort resulted in derivatives **22**, **23**, **24**, and **25**, featuring a piperidinobenzamide motif. While the scaffold hopping yielded new TCA1 analogs with enhanced activity against *Mtb* compared to TCA1, the derivatives retained unfavorable drug-like properties as seen in TCA1, including microsomal instability and poor bioavailability. (b) Comparative analysis of the binding mode of TCA1 (PDB ID: 4KW5) and analog **22**. The binding mode of **22** was suggested by docking **22** into the DprE1 active site (PDB ID: 4KW5, DprE1 complexed with TCA1) using the CDOCKER protocol. The top-scored binding pose of **22** closely resembled that of TCA1. Critical H-bonds between the ligand and the DprE1 active site are depicted as gray dashed straight lines. Hydrophobic interactions are illustrated as gray dashed circular lines.

Researchers applied scaffold hopping to replace the benzothiazole in TCA1 with a benzamide motif containing various aliphatic and cyclic amines to introduce an additional hydrogen bond acceptor. Among the series of 16 compounds, compound **22** revealed enhanced activity against *Mtb* and reduced cytotoxicity compared to TCA1 (**22**: MIC *Mtb* H_37_Rv = 0.047 μM; IC_50_ Vero >149 μM vs MIC TCA1: *Mtb* H_37_Rv = 1.27 μM; IC_50_ Vero = 85 μM, respectively; Figure 12a). Compound **22** served as a template for subsequent docking studies. The docking studies with **22** into the active site of DprE1 (PDB ID: 4KW5) revealed binding properties similar to those of TCA1. Additionally, the carbonyl group of the amide bond linking the phenyl and piperidine moieties replaced the original benzothiazole in the arylamide region (Figure 12b). This led to the formation of an extra hydrogen bond with Tyr60, aligning with the previous hypothesis of Wang et al.^110^

Further SAR studies on derivative **22** targeted the arylamide moiety, where fluorine substitution on the phenyl ring enhanced π–π stackings with the hydrophobic pocket of DprE1, resulting in compound **23** (Figure 12a). Derivative **23** exhibited similar activity under *in vitro* conditions against *Mtb* compared to **22** (**23**: MIC *Mtb* H_37_Rv < 0.036 μM vs **22**: MIC *Mtb* H_37_Rv = 0.047 μM), while retaining a favorable cytotoxicity profile as measured on Vero cell line (IC_50_ > 143 μM). Given that the imido ester side chain poses a notable metabolic liability, the derivatization of the most promising compounds **22** and **23** was conducted on the terminal imido ester moiety. From a series of 17 compounds, derivatives **24** (MIC *Mtb* H_37_Rv = 0.46 μM) and **25** (MIC *Mtb* H_37_Rv = 0.07 μM), structural analogues of **22** and **23**, respectively, were identified as promising candidates (Figure 12a). Both **24** and **25** contain an imido methyl ester group instead of the original imido ethyl ester present in TCA1 and **22**. Additionally, compounds **22**, **23**, **24**, and **25** were effective against two XDR-TB strains, with MIC values ranging from 0.07 to 0.58 μM. Furthermore, compounds **22**, **23**, and **24** displayed sustained activity against *Mtb* strains resistant to BTZs (MIC ranged from 0.14 to 1.16 μM vs 3.28 μM for TCA1), suggesting a low risk of cross-resistance and indirectly indicating that these TCA1 derivatives retain noncovalent DprE1 inhibition. In *in vitro* cardiotoxicity assessment, none of these compounds demonstrated higher hERG channel inhibition than TCA1 (**22**, **23**, **24** and **25**: hERG IC_50_ values > 22.6 μM vs TCA1 hERG IC_50_ = 18.3 μM). Compounds **22**, **23**, **24**, and **25** were further assessed in a macrophage model of acute TB infection, demonstrating intracellular efficacy with log_10_ CFU reduction values ranging from 0.75 to 1.34 at a concentration of 10 μg/mL over 3 days, comparable to TCA1, which achieved a 1.16 log_10_ CFU reduction under identical experimental conditions. Building on these findings, researchers evaluated compound **24** in a murine model of acute TB infection. At a dose of 100 mg/kg over 3 weeks, compound **24** achieved a 2.02 log_10_ CFU reduction, albeit less effective than TCA1 (2.86 log_10_ CFU reduction under the same conditions), still demonstrated promising *in vivo* activity.^110^

Finally, the metabolic stability of compounds **22**, **23**, **24**, and **25** was determined using MLMs and HLMs. In MLMs, all derivatives displayed moderate metabolic turnover, with a shorter elimination half-life than TCA1. Notably, compound **25** exhibited an elimination half-life approximately 7-times shorter than that of TCA1 (**19** *T*_1/2_ = 12.5 min vs TCA1 *T*_1/2_ = 85.3 min). Conversely, in HLMs, all derivatives showed improved elimination half-life compared to TCA1 (*T*_1/2_ ranging from 20.3 to 50.8 min vs TCA1 *T*_1/2_ = 5.97 min). Unfortunately, derivatives **23** and **24** demonstrated a very low oral bioavailability *in vivo* (**23**: *F* = 2.3%; **24**: *F* = 7.9% vs TCA1: *F* = 42.6%), limiting their translational potential.^110^

The reduction in MIC in *M. bovis* strain overexpressing *Mt*-DprE1 indicates that these TCA1 derivatives maintain DprE1 inhibition.^110^

Pyrrolothiadiazole **26**, identified within the anti-TB compound library released by GlaxoSmithKline (GSK) exhibits *Mtb* MIC_90_ H_37_Rv value of 4.0 μM and a DprE1 pIC_50_ of 7.3 (Figure 13).^102^ Compound **26** was characterized as a noncovalent DprE1 inhibitor through HTS, as evidenced by its diminished activity against an *Mtb* strain overexpressing DprE1.^111^ However, compound **26** exhibited unfavorable physicochemical properties, in particular very low solubility (CLND solubility <1 μM). Additionally, the pyrrolothiadiazole backbone present in **26** poses a potential metabolic and cytotoxic liability.^112^

**Figure 13:**
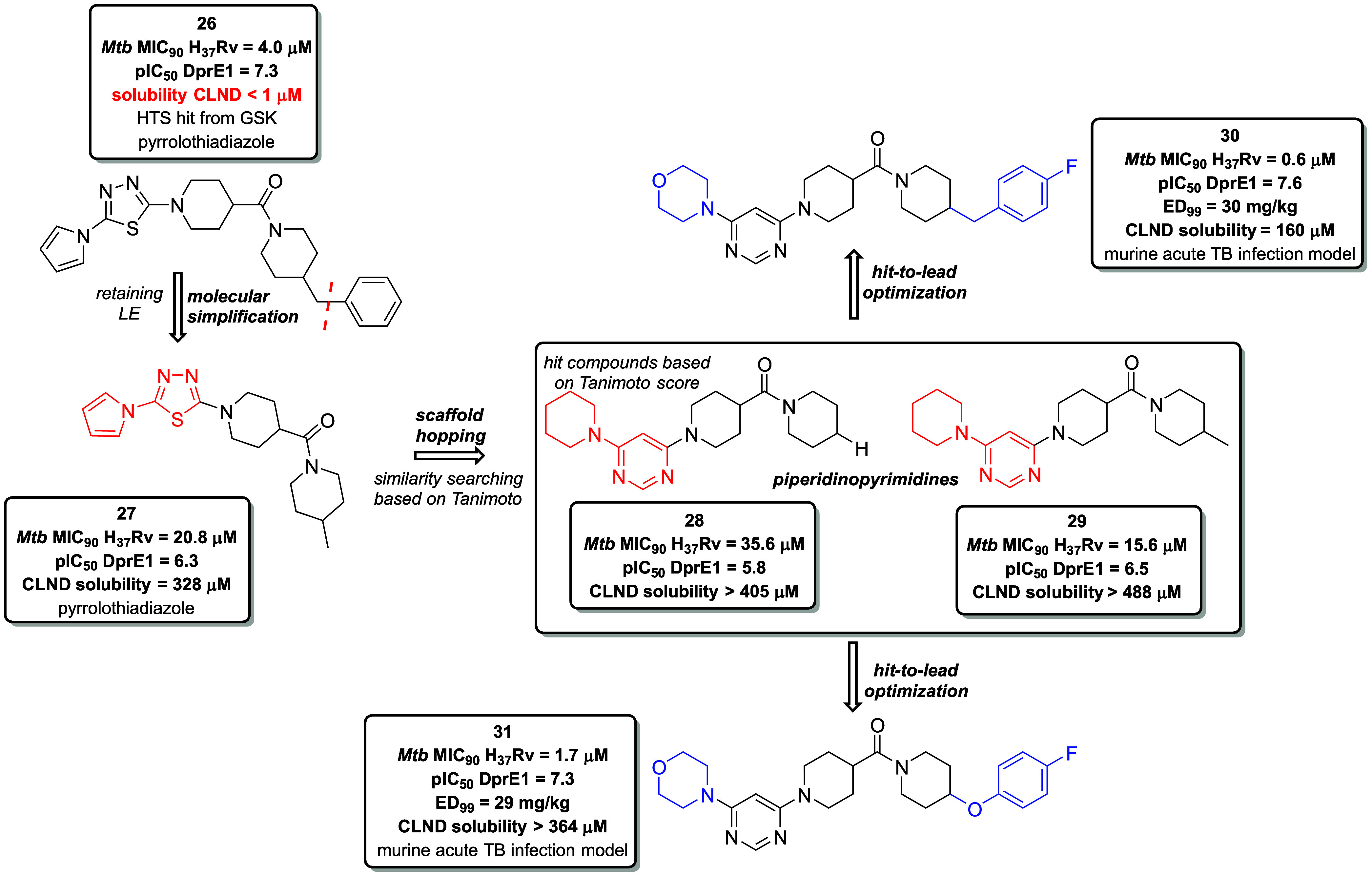
Rationally driven hit-to-lead protocol performed by Borthwick et al.^113^ leading to the discovery of a novel class of morpholinopyrimidines as DprE1 inhibitors. 4° Scaffold hopping, computer-aided strategy based on the similarity searching within the in-house library of anti-TB compounds, was employed to address the physicochemical properties limitation and potential metabolic liabilities of hit compound **26**, which featured pyrrolothiadiazole scaffold, identified by GSK. This effort led to the discovery of a completely new class of piperidinopyrimidines inhibiting DprE1. Hit-to-lead optimization of **28** and **29** resulted in morpholinopyrimidine derivatives **30** and **31**, both efficient in a murine model of acute TB infection. Both derivatives **30** and **31** displayed significantly improved solubility compared to the hit **26** from GSK.

To address the nonoptimal physicochemical profile of molecule **26**, Borthwick et al. applied scaffold hopping as a part of their hit-to-lead optimization. This optimization process began by truncating the original benzyl moiety found in **26**, substituting it with a methyl at the 4-position of the terminal *N*-acylpiperidine, affording derivative **27** formation that retains acceptable ligand efficiency (Figure 13). Scaffold hopping was guided by similarity searching for complementary scaffolds to the original simplified pyrrolothiadiazole **27** within the in-house collection, utilizing the Tanimoto score as the similarity descriptor. As a result of this effort, hit structures **28** (*Mtb* MIC_90_ H_37_Rv = 35.6 μM; DprE1 pIC_50_ = 5.8; Figure 13) and **29** (*Mtb* MIC_90_ H_37_Rv = 15.6 μM; DprE1 pIC_50_ = 6.5; Figure 13) were identified, bearing a piperidinylpyrimidine core. Although **28** and **29** were less active than **26**, they exhibited significantly improved solubility (CLND solubility >405 μM and >488 μM, respectively).^113^

In the subsequent stages of hit-to-lead optimization, substructures **28** and **29** were modified to enhance their biological activity against *Mtb*. The newly discovered compounds, distinguished by their substitution at 4-position on the terminal *N*-acylpiperidine, were optimized with the priority of maintaining their superior physicochemical properties compared to **26**. Modifications of compounds **28** and **29** included the replacement of the piperidine moiety attached to the pyrimidine core, along with the isosteric introduction of either a 4-fluorobenzyl (derivative **30**; Figure 13) or a 4-fluorophenoxy (derivative **31**; Figure 13) substituent at the 4-position of the terminal *N*-acylpiperidine. Such modifications led to the development of a novel class of morpholinopyrimidines as potent, noncovalent DprE1 inhibitors. Derivatives **30** and **31** displayed significantly improved antimycobacterial activity toward *Mtb* H_37_Rv and the equipotent activity against DprE1 to the parent compound **26**, while also showing low cytotoxicity for HepG2 cells. Additionally, compounds **30** and **31** exhibited notably improved aqueous solubility compared to **26**. Further evaluation of **30** and **31** in an acute toxicity model in C57Bl/6 mice yielded promising ED_99_ values (the dosage required to reduce a pulmonary mycobacterial load by 99% at day 9 postinfection relative to the untreated group) of 30 and 29 mg/kg, respectively, after 8 days of daily oral administration. However, compounds **30** and **31** revealed contrasting trends in their microsomal stability against both MLMs and HLMs. In microsomal fraction stability evaluation with HLMs, **30** showed moderate to high microsomal clearance, while microsomal clearance of **31**, bearing 4-fluorophenoxy moiety, was significantly reduced. *In vivo* PK studies in mice further indicated that both derivatives had short half-lives in MLMs (**30**: *T*_1/2_ = 27 min; **31**: *T*_1/2_ = 60 min) but displayed excellent oral bioavailability. A comparative analysis of the binding modes between the original pyrrolothiadiazole **26** and lead compounds **30** and **31** was not conducted. Since then, the binding mode of **30** and **31** and active site of DprE1 remained elusive.^113^

## Conclusion

TB remains an urgent global health challenge, further exacerbated by the emergence of drug-resistant *Mtb* strains. This ongoing crisis highlights the critical need for novel therapeutic strategies. In this context, scaffold hopping has emerged as a promising approach in TB drug discovery, offering potential to overcome key limitations of current anti-TB agents, including resistance, toxicity, and poor pharmacokinetic profiles.

### How Scaffold Hopping Supports Medicinal Chemistry in TB Drug Discovery?

Scaffold hopping involves structural modifications within the core of known bioactive compounds to yield new chemotypes while preserving biological activity. This strategy enables the design of drug candidates with improved pharmacological profiles, including enhanced potency, reduced toxicity, resistance circumvention, and optimized pharmacokinetic properties. From a medicinal chemistry standpoint, scaffold hopping provides a unique opportunity to address various drawbacks of existing lead compounds such as poor solubility, synthetic feasibility, high toxicity, acquired resistance, and metabolic liability, without the need for repeated and costly screening efforts. Moreover, it facilitates navigation around IP barriers and allows for expansion of the IP space.

### Scaffold Hopping by Degree: Advantages and Unmet Challenges

1° scaffold hopping has proven to be a widely used and relatively successful approach in TB drug discovery, particularly in optimizing known clinical candidates like Q203, TBA7371, and BTZs derivatives. It enables subtle heterocyclic modifications that maintain key pharmacophoric features and improve physicochemical properties, often yielding candidates with improved solubility, metabolic stability, or reduced toxicity. However, evidence from several studies, including derivatives like ND-1543 ^49^ and some PBTZ-169 analogs,^60^ demonstrates that despite strong *in vitro* activity and pharmacophore retention, modest structural changes can still lead to poor *in vivo* efficacy or unfavorable pharmacokinetic profiles. Moreover, the limited structural novelty in 1° hops often translates to marginal IP gains and their contribution to overcoming drug resistance remains constrained unless combined with other optimization strategies.

2° scaffold hopping involves conformational restriction (ring closure) or flexibility enhancing (ring opening), with impactful results seen in compounds like OTB-658^71^ or coumestan-based PKS13 inhibitors.^73,75^ The ring closure strategy applied on a class of oxazolidinone-based ATBs led to the discovery of OTB-658, effectively enhancing selectivity against bacterial ribosome and significantly reducing adverse effects such as mitochondrial toxicity and inhibition of monoamine oxidases observed in LNZ and sutezolid.^71^ In the class of PKS13 inhibitors derived from the parent compounds TAM1 ^74^ and TAM16,^72^ application of structural rigidification resulted in the new coumestan analogs with improved metabolic stability, oral bioavailability, and sustained *in vivo* efficacy in a mouse model of acute TB infection.^73^ ,^75^ Conversely, ring opening, such as the conversion of BTZs to DNB analogs, has yielded derivatives with better pharmacokinetic profiles but occasionally at the cost of reduced potency or altered safety profiles.^78^ Importantly, the impact of such changes on target binding and intracellular activity is difficult to predict without robust structural data, highlighting a major limitation of 2° scaffold hopping in the absence of structural biology support.

3° scaffold hopping addresses challenges of peptide drugs such as metabolic liability, poor membrane permeability, and selectivity issues. In the context of TB, this strategy has enabled the design of proteasome inhibitors selective for *Mtb* 20S over Hu 20S, primarily by optimizing interactions of peptide backbones with the S1 and S3 binding pockets in *Mtb* 20S. While compounds like DPLG-2 ^103,104^ and syringolin derivatives^98^ showed excellent selectivity and whole-cell activity in surrogate models, translation into *Mtb* remains elusive. Furthermore, the still high molecular weight and other limitations associated with the current peptide-based *Mtb* 20S inhibitors, emerging from the application of peptidomimetics, could potentially result in undesired off-target effects and poor oral bioavailability, highlighting persistent hurdles. Despite significant promise, peptidomimetics still require careful validation in full infection models and improved strategies to overcome permeability and systemic stability issues.

4° scaffold hopping involves the most dramatic structural transformations and is primarily guided by in silico methods. Its use in TB drug discovery remains rare and is often associated with high failure rates. Although this approach enables the design of compounds with novel chemotypes, many resulting derivatives (e.g., TCA1 analogs) retain liabilities such as metabolic instability or exhibit toxicity profiles similar to those of their parent scaffolds. Moreover, often unclear binding modes limit mechanistic insights. Overreliance on docking and VS, especially in the absence of high-quality structural information, can lead to misleading predictions, making this degree of scaffold hopping a high-risk strategy that requires integration with experimental validation.

### Integration with Computational Tools and the Need for Rational Design

Scaffold hopping has proven to be a versatile and innovative approach that benefits significantly from integration with computational methods. Whereas traditional applications often rely on the intuition of medicinal chemists, *in silico* tools allow for a more systematic exploration of chemical space. Advanced modeling techniques, including shape matching, pharmacophore modeling, fragment replacement, and similarity searching, play a crucial role in prioritizing scaffold modifications. Notably, VS (whether LBVS or SBVS) enables the selection of scaffolds based on predicted binding affinity. Molecular docking, the cornerstone of SBVS, predicts binding poses and interaction strengths, facilitating rational molecular design and scaffold optimization.

These computer-aided tools are especially valuable for higher-degree scaffold hopping (e.g., 3° and 4°), which is almost exclusively driven by *in silico* methods. The classification system proposed by Sun and co-workers, dividing scaffold hopping into four degrees, provides a practical framework for systematic implementation. It enables targeted modifications of molecular backbones, detailed analysis of structural features, and evaluation of how scaffold changes impact ligand–protein binding interactions. Future developments in AI-driven drug design and machine learning are likely to further enhance the predictive power and efficiency of scaffold hopping strategies, accelerating the discovery of novel anti-TB compounds.

### Final Remarks

In conclusion, scaffold hopping represents a well-established strategy in addressing the multifaceted challenges of TB drug discovery. Its application has led to the identification of promising new candidates with the potential to reshape TB therapy, particularly in combating resistant *Mtb* strains. Continued investment in scaffold hopping, supported by advances in computational and synthetic chemistry, is poised to unlock new therapeutic avenues and bring us closer to the goals of the WHÓs “End TB strategy.” By fostering innovation and addressing unmet clinical needs, scaffold hopping holds great promise for transforming TB care and reducing the global burden of this devastating disease.

## Abbreviations used

ACP: acyl carrier protein domain;
BDQ: bedaquiline
BM scaffold: Bemis-Murcko scaffold
BTZs: benzothiazinones
CFU: colony forming unit
CLND: chemiluminescent nitrogen detection
DNBs: dinitrobenzamides
DPA: decaprenylphosphoryl-β-d-arabinose
DprE1: decaprenylphosphoryl-β-d-ribose-2́-epimerase
DR-TB: drug-resistant TB
DS-TB: drug susceptible TB
ED_99_: dosage required to reduce a pulmonary mycobacterial load by 99%
EMA: European Medicines Agency
EMB: ethambutol
ETC: electron transport chain
FAS: fatty acid synthesis
GSK: GlaxoSmithKline
HierS: hierarchical scaffold clustering using topological chemical graphs
HLMs: human liver microsomes
HTS: high-throughput screening
Hu 20S: human proteasome
Hu c-20S: human constitutive proteasome
Hu i-20S: human immunoproteasome
INH: isoniazid
IP: intellectual property
IR-TB: isoniazid-resistant TB
LBVS: ligand-based virtual screening
LNZ: linezolid
MAO: monoamine oxidase
MmpL3: mycobacterial membrane protein large 3
MPS: mitochondrial protein synthesis
*Mtb*: *Mycobacterium tuberculosis*
PDE: phosphodiesterase
PKS: polyketide synthase
PMF: proton motive force
pre-XDR-TB: pre-extensively resistant TB
PZA: pyrazinamide
RIF: rifampicin
RND: resistance, nodulation, and division
RNS: reactive nitrogen species
RR-TB: rifampicin-resistant TB
SBVS: structure-based virtual screening;
SI: selectivity index
TB: tuberculosis
TE: thioesterase domain
TMM: trehalose monomycolate
WHO: World Health Organization
XDR-TB: extensively resistant TB.
